# Identification of QTLs linked to bioactive flavonoids and glycosides in the apricot fruit (*Prunus armeniaca* L.)

**DOI:** 10.1186/s12864-026-12989-0

**Published:** 2026-05-30

**Authors:** Germán Ortuño-Hernández, Jesús Guillamón Guillamón, Raquel Sánchez-Pérez, Álvaro Delgado, José Enrique Yuste, David Ruiz, Pedro Martínez-Gómez, Juan Alfonso Salazar

**Affiliations:** 1 Fruit Breeding Group, Department of Plant Breeding, CEBAS-CSIC (Centro de Edafología y Biología Aplicada del Segura-Consejo Superior de Investigaciones Científicas), Campus Universitario Espinardo, Murcia, 30100 Spain; 2 Metabolomics Platform of CEBAS-CSIC, Campus Universitario Espinardo, Murcia, 30100 Spain

**Keywords:** Apricot, Flavonols, Flavan-3-ols, Glycosides, Phenolic compounds, *Prunus armeniaca* L., QTLs, UPLC-QToF-MS/MS

## Abstract

Apricots are highly valued for their nutritional and functional properties, largely due to their rich content of bioactive compounds, particularly flavonoids, phenolic acids, and glycosides. These secondary metabolites are associated with antioxidant, anti-inflammatory, and antimicrobial activities, as well as contributing to fruit flavor, aroma, and overall quality. This study investigated the genetic basis of flavonoid and glycoside accumulation by integrating untargeted metabolomics (UPLC-QToF-MS/MS) with QTL mapping in two segregating apricot populations: ‘Bergeron’ × ‘Currot’ and ‘Goldrich’ × ‘Currot’. Five flavonoids (catechin, epicatechin, myricitrin, quercetin, and rutin), three phenolic acids (coumaric, caffeic, and ferulic acids), and five glycosides (kiwiionoside, neryl arabinofuranosyl-glucoside, vanilloyl glucose, zizybeoside I, and 3-hydroxy-beta-ionol 3-[glucosyl-(1- > 6)-glucoside]) were tentatively identified from metabolite features showing significant differences between parental genotypes (ANOVA, *P* < 0.05) and subsequently detected across parents and seedlings. QTL mapping identified loci associated with epicatechin on linkage group 1 (LG1), myricitrin and rutin on LG7, and vanilloyl glucose on LG4. In contrast, zizybeoside I exhibited polygenic genomic architecture, with associations distributed across several chromosomes, predominantly on LG2, LG3, and LG5. These findings highlight the genetic loci associated with flavonoid and glycoside biosynthesis, providing valuable insights into marker-assisted selection (MAS) strategies in apricot breeding programs aimed at enhancing the nutritional and functional value of this species.

**Supplementary Information:**

The online version contains supplementary material available at 10.1186/s12864-026-12989-0.

## Introduction

Apricot (*Prunus armeniaca* L.) is a highly valued stone fruit known for its rich flavor and nutritional profile, playing an important role in human diets across cultures due to its content of essential vitamins, minerals, and bioactive compounds [1]. Globally, it ranks as the third most produced stone fruit, with an annual yield of 3.86 Mt, following peach/nectarine (26.35 Mt) and plum (12.39 Mt) [2]. Apricot cultivation is widely distributed, with Turkey and Uzbekistan being the leading producers, while Spain is the main exporter, especially in terms of fresh fruit, followed by Turkey, Uzbekistan, and Italy, all of them benefiting from suitable climatic and soil conditions. Beyond its traditional consumption as fresh or dried fruit, apricot has increasingly gained recognition as a health-promoting food, reinforcing its importance in fruit production and nutrition [3].

Among the secondary metabolites present in apricot fruit, flavonoids, organic acids, and glycosides stand out for their biological relevance and contributions to its quality. Flavonoids are a diverse family of phenolic compounds that have been extensively studied for their antioxidant, anti-inflammatory, and cardioprotective properties, as well as their potential role in cancer prevention [4–7]. They are classified into subgroups based on chemical structure, including flavonols (quercetin, myricetin), flavan-3-ols (catechin, epicatechin), flavones (apigenin, luteolin), isoflavones (genistein, daidzein), flavanones (naringenin, hesperidin), anthocyanidins (cyanidin, delphinidin), and chalcones, which act as biosynthetic precursors [8–13]. Organic acids, such as citric, malic, and tartaric acids, are key determinants of acidity and flavor, and they also participate in energy production and plant stress responses [14, 15]. Glycosides, which include phenolic and terpenoid glycosides, contribute to fruit aroma, sweetness, and functional properties. Many glycosides are bioactive, conferring antioxidant, antimicrobial, and anti-inflammatory activities that further enhance apricot’s health-promoting potential [16, 17]. Together, these metabolites contribute distinctively to plant physiological processes and exert diverse health-promoting effects in humans, underscoring the importance of apricot as a rich source of bioactive compounds for nutritional and functional purposes.

The biosynthesis of flavonoids, organic acids, and glycosides involves distinct yet interconnected metabolic pathways. Flavonoids and many glycosides are products of the phenylpropanoid pathway, whereas organic acids are linked to the tricarboxylic acid cycle [18]. Glycosylation is a key modification process that influences metabolite solubility, stability, and bioactivity [19]. This biochemical complexity underpins the need for genetic studies aimed at elucidating the regulation of secondary metabolism in apricot fruits.

Identifying quantitative trait loci (QTLs) associated with specific metabolites is a powerful approach to understanding the genetic basis of secondary metabolism. Such knowledge facilitates the development of molecular markers for marker-assisted selection (MAS), accelerating breeding strategies. In apricot, most reported QTLs are associated with phenological and agronomic traits, including flowering and ripening times, pistil abortion, and fruit quality attributes such as firmness, color, and acidity [20–22]. However, QTL studies specifically targeting metabolites remain scarce. Among the limited work available, research has focused primarily on organic acids [23].

Comparative insights from related *Prunus* species have revealed QTLs for both flavonoids and organic acids, underscoring the conserved nature of metabolic regulation across the genus. For instance, QTLs associated with flavonoids have been described in peach (*Prunus persica* L. Batsch) [24], sweet cherry (*Prunus avium* L.) [25], and Asian plum (*Prunus salicina* Lindl.) fruits [26]. Likewise, QTLs for organic acids and other compounds have been reported in peach and sweet cherry [27, 28]. More recently, QTLs related with the content of glycosylated derivatives have been identified in peach and sweet cherry [29, 30], reinforcing the relevance of glycosylation in secondary metabolism. These findings highlight the need to expand metabolite-focused QTL studies in apricot, where genomic resources are now increasingly available.

Technological advances in untargeted metabolomics have revolutionized the study of plant secondary metabolism. Ultra-Performance Liquid Chromatography coupled with Quadrupole Time-of-Flight Mass Spectrometry (UPLC-QToF-MS/MS) enables the detection and quantification of a broad spectrum of metabolites, allowing detailed metabolic profiling [31]. When combined with genetic mapping, this approach provides unprecedented resolution in linking metabolic variation to underlying loci.

The combined application of secondary metabolite profiling and genetic mapping remains costly, as it requires the analysis of a large number of genotypes within segregating populations, and is therefore rarely applied. As mentioned above, similar approaches have been used in related species, integrating genomics, targeted metabolomics, and molecular biology to explain metabolic variation. However, in this work we applied untargeted metabolomics. In apricot, such an integrative approach has not been previously reported, making this study a pioneering effort in uncovering associations between metabolic traits and genetic loci in this species.

The present research integrates untargeted metabolomics with QTL mapping to dissect the genetic basis of flavonoid, phenolic acid, and glycoside accumulation in apricot fruits during maturation. By characterizing the diversity of these compounds and identifying the genomic regions associated with their biosynthesis, we aim to establish a foundation for MAS strategies directed at improving both the sensory and functional qualities of fruit in apricot. Ultimately, this research contributes to a deeper understanding of secondary metabolism in *Prunus* spp. and supports the development of improved fruit cultivars with enhanced nutritional and commercial value.

## Material and methods

### Plant material and experimental design

The plant material evaluated in 2023 consisted of two F1 populations: ‘Bergeron’ × ‘Currot’ (‘B × C’, *n* = 134) and ‘Goldrich’ × ‘Currot’ (‘G × C’, *n* = 159). Both populations were established in 2009 at the CEBAS-CSIC experimental orchard, located in Cieza-Calasparra, Murcia, Spain (37° N, 1° W, 450 m altitude). The progenies of the two families analyzed in this study were established on their own roots. Due to the large number of genotypes evaluated, individuals from both populations were planted under a high-density system, with a spacing of 5 × 1.5 m. Crop management included fertigation via drip irrigation. Apricot trees were trained using an open-center system and subjected to light winter pruning. These populations were specifically developed to maximize phenotypic variability in traits related to phenology and fruit quality (Table 1) [32]. The harvest criterion used in this trial was based on the transition in fruit color and field firmness, applied to 100 genotypes from each population. In addition to being segregating populations for pomological traits, they also segregate for nutritional profile, including both primary and secondary metabolites, as reported in a previous study that laid the foundation for this work [33].

**Table 1:** Characteristics of the parental lines used in the crosses (‘Bergeron’ × ‘Currot’ and ‘Goldrich’ × ‘Currot’) within the breeding program

**Cultivar** **(crosses)**	**‘Bergeron’**	**X**	**‘Currot’**	**‘Goldrich’**	**X**	**‘Currot’**
Chill requirements	1200 CU		600 CU	1100 CU		600 CU
Flowering time	Late		Early	Late		Early
Ripening time	Late		Early	Mid-late		Early
Skin color	Light Orange		Light Yellow/Pink	Orange		Light Yellow/Pink
Self-compatibility	Self-compatible		Self-compatible	Self-incompatible		Self-compatible
Sharka resistance (PPV-D)	No		No	Yes		No
Fruit weight	Medium		Low	High		Low
Acidity	Medium		Low	High		Low
Ethylene production	Very low		High	Low		High
Firmness	Medium–high		Medium	High		Medium

### Pomological traits analysis

Twelve fruits from each seedling were harvested to evaluate pomological traits related to color, including the chlorophyll index (I_AD_), skin color, blush color, flesh color, and percentage of blush coverage. The chlorophyll index was measured using a DA-meter (Sinteléia, Bologna, Italy), a portable Vis–NIR spectrometer that estimates fruit maturity based on I_AD_. Fruit color was assessed using a Minolta colorimeter (CR-300; Minolta, Ramsey, NJ, USA), with three measurements taken on both the skin and the flesh after calibration with a white porcelain reference plate. The CIELAB color space was used to quantify three coordinates: L* (lightness), a* (red-green), and b* (yellow-blue). Additionally, the Hue angle (hº = arctangent (b*/a*)) was calculated [34], with values between 85 and 95 indicating a yellow hue, values from 70 to 80 signifying an orange hue, and values below 70 representing a reddish hue. Additionally, the percentage of fruit blush coverage was determined visually.

### Untargeted UPLC-QToF-MS/MS analysis

For each seedling, a mix was prepared by pooling 12 fruits. This mix, including the edible portion of the fruit (exocarp and mesocarp), was lyophilized for subsequent untargeted UPLC-QToF-MS/MS analysis, focusing on the 60 most contrasting genotypes from each population. The selection criterion was based on fruit color, using hue angle (h°) values to define phenotypic extremes within the lowest 30% (≤ P30) and highest 30% (≥ P70). Accordingly, 30 genotypes with the highest h° values (yellow phenotypes) and 30 with the lowest h° values (orange phenotypes) were selected. Three technical replicates of the lyophilized mixture were prepared, with each replicate weighing 50 mg. These samples were extracted using 1 mL of an HPLC-grade methanol/water mixture (80:20, v/v). The extraction process involved mechanical agitation with a vortex, sonication in three 30-s intervals, and centrifugation at 13,000 × g for 10 min, following the method described [35]. Glipizide (Sigma) was added at a concentration of 0.1 μg/mL as an internal standard. The resulting extracts were filtered through a 13 mm PVDF syringe filter (0.22 μm, Millipore) prior to analysis.

Chemical profiling was performed using UPLC-QToF-MS/MS, combining a Waters ACQUITY UPLC I-Class System (Waters Corporation, Milford, MA, USA) with a Bruker Daltonics QToF-MS mass spectrometer (maXis impact series, resolution ≥ 55,000 FWHM, Bruker Daltonics, Bremen, Germany). Chromatographic separation was achieved on an HSS T3 C18 column (100 × 2.1 mm, 1.8 μm particle size, Waters Corporation) at a flow rate of 0.3 mL/min. Mobile phases consisted of water with 0.01% formic acid (pH ~ 3.2) (PanReac AppliChem, Barcelona, Spain) as phase A and acetonitrile with 0.01% formic acid (J. T. Baker, New Jersey, USA) as phase B. Gradient program was a modified version [36]. It started at 10% mobile phase B, gradually increased to 90%, held for a brief period, then rapidly decreased back to 10% within 10 s and remained at this level until the chromatogram was completed. Column temperature was maintained at 40 °C, and the drying temperature was set to 200 °C. Nitrogen was used as both the desolvation gas (8 L/min) and the nebulizing gas (2.0 bar). The source voltage was set to 4.5 kV in positive electrospray ionization mode (ESI +). High-resolution MS data were acquired over an m/z range of 45–1200 Da using broadband collision-induced dissociation (bbCID) at 24 eV for ESI (+). External calibration was performed before each sequence using a 10 mM sodium formate solution delivered by a KNAUER Smartline Pump 100 equipped with a pressure sensor (KNAUER, Berlin, Germany). Calibration solution consisted of 0.5 mL of formic acid, 1.0 mL of 1.0 M sodium hydroxide, and a 1:1 (v/v) isopropanol/Milli-Q water mixture.

### Metabolite tentatively identified from Untargeted UPLC QToF MS/MS

A total of thirteen compounds out of 585 significant features were tentatively identified following the metabolite identification protocol established [33]. These metabolites included five flavonoids, three phenolic acids, and five glycosides. The flavonoid fraction comprised catechin and epicatechin, both belonging to the flavanol subclass, and myricitrin, quercetin, and rutin, classified as flavonols according to KEGG. Myricitrin and rutin are glycosylated derivatives of myricetin and quercetin, respectively, contributing to both the antioxidant capacity and the functional value of apricot fruit. The phenolic acids identified were coumaric acid, caffeic acid, and ferulic acid, all members of the hydroxycinnamic acid subclass. In addition, five glycosides were detected: kiwiionoside (G1), neryl arabinofuranosyl-glucoside (G2), vanilloyl glucose (G3), zizybeoside I (G4), and 3-hydroxy-beta-ionol 3-[glucosyl-(1 → 6)-glucoside] (G5). These belong to the subclass of glycosylated metabolites, specifically linked to phenylpropanoid derivatives (G3) or terpenoid-derived glycosides (G1, G2, G4, and G5).

### Marker-trait association and QTL analysis

Marker-trait association analyses between SNPs and pomological traits, as well as metabolite data, were performed using TASSEL v5 [37]. The SNP dataset from the ‘B × C’ and ‘G × C’ populations used in this study has been previously published [22]. A General Linear Model (GLM) was employed for these analyses, combining quantitative phenotypic data with genotypic information and principal component analysis (PCA). Manhattan plots were generated for the most relevant metabolites to visually display the significance of SNP associations across the genome for each selected metabolite.

QTL identification was performed by integrating metabolomic and pomological trait data with genetic maps derived from SNP genotyping [22], using the MAPQTL v7 software [38]. To highlight the most significant QTLs, both parametric (interval mapping) and non-parametric (Kruskal–Wallis) approaches were used. In addition, multiple QTL mapping (MQM) analysis was performed using the most significant SNP as a cofactor to refine QTL detection. LOD (logarithm of odds) thresholds for significance were determined individually for each trait through 1,000 permutation tests (*P* = 0.05), using the “Permutation Test” function to ensure robust statistical validation.

### Functional annotation of candidate genes

Functional annotation of candidate genes was carried out using eggNOG-mapper (version 2.1.13) [39], an orthology-based tool that facilitates the assignment of functional information by transferring annotations from well-characterized homologous genes. Protein sequences were searched against the eggNOG database using DIAMOND blastp in sensitive and iterative modes [40]. Annotations were assigned based on orthology relationships, applying an E-value threshold of 1 × 10⁻^3^ and retaining up to the three best hits per query. Alignment coverage for both query and subject sequences was automatically evaluated by eggNOG-mapper to exclude low-confidence matches.

### Data analysis

Pearson correlation analyses were conducted to evaluate the relationships between metabolomic and pomological data. The selection of metabolite groups for the correlation analyses was based on their well-established biological relevance to the traits studied. Specifically, flavonoids such as flavonols and flavan-3-ols were selected due to their involvement in the phenylpropanoid pathway. Although these compounds are not the primary pigments responsible for fruit coloration, they share biosynthetic pathways with other phenolic compounds that contribute to color development. Therefore, their evaluation in relation to color-related traits may provide insights into coordinated metabolic regulation. Likewise, phenolic acids and their glycosylated derivatives are key intermediates and end-products within the phenylpropanoid pathway, playing an important role in determining fruit nutritional and functional quality. The significance levels were assessed at thresholds of 0.05 and 0.01. All statistical analyses were performed using INFOSTAT v18 software (National University of Córdoba, Argentina).

## Results

### Fruit color and flavonoids

**Fig. 1:**
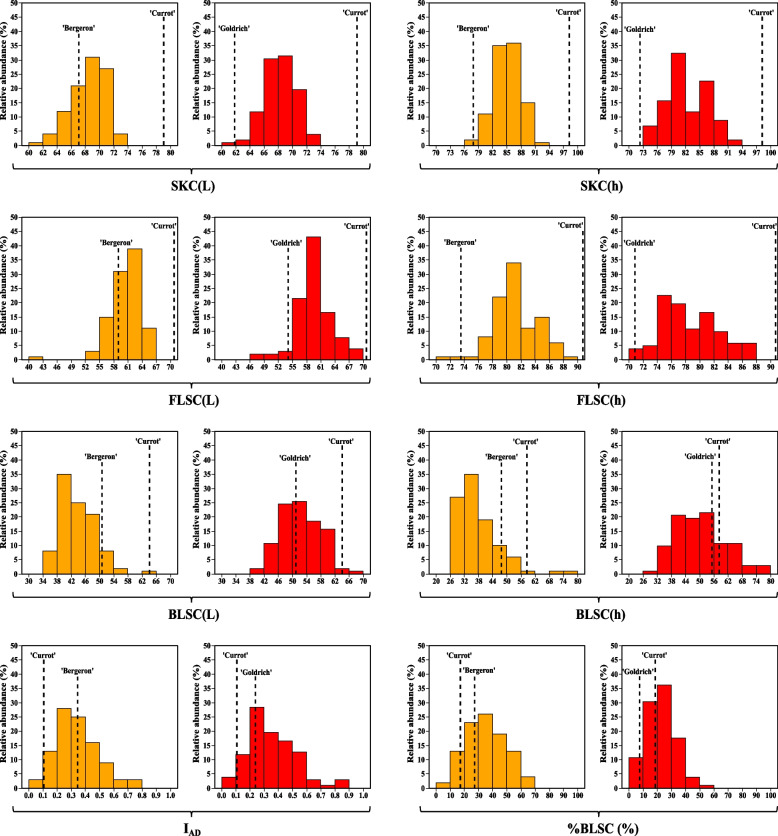
Frequency histograms of traits related to color in the ‘Bergeron’ × ‘Currot’ (orange bars) and ‘Goldrich × ‘Currot’ (red bars) populations: skin color (SKC), flesh color (FLSC), and blush color (BLSC), assessed based on two components: lightness (L) and hue (h), percentage of fruit blush (%BLSC), and chlorophyll index (I_AD_)

Phenotypic data on color-related pomological traits were summarized by frequency histograms for two segregating apricot populations, evaluated in terms of lightness (L) and hue (hº) (Fig. 1). For skin and flesh color (SKC and FLSC), the data distributions were bounded by the parental values in both populations, showing very similar patterns in the lightness component. For hue (hº), the ‘G × C’ population exhibited a tendency toward deeper orange colors, with values below 80, whereas the ‘B × C’ population displayed a yellowish-orange hue, with values around 85, probably due to the genetic background of the parents ‘Currot’ (light yellow) and ‘Goldrich’ (intense orange).

For traits related to fruit blush (BLSC), both populations showed data distributions below the maternal cultivars (‘Bergeron’ and ‘Goldrich’) in terms of hue, indicating a more intense red blush in the segregating individuals. In contrast, for blush lightness (BLSC(L)), the ‘B × C’ population predominantly exhibited lightness values lower than 'Bergeron' (< 50), while in ‘G × C’, most data were distributed between the parental values, with the lower limit being ‘Goldrich’ at 50 and ‘Currot’ at approximately 65.

Histograms of blush percentage indicated an increase in blush coverage in individuals from both populations compared to their respective parental lines. In the ‘B × C’ population, seedlings with more than 50% blush coverage were observed, while in the ‘G × C’ population, a substantial number of individuals displayed 20–30% blush coverage, probably due to the low covered observed in the parental ‘Goldrich’.

Regarding I_AD_, although individuals from both populations exhibited values exceeding those of the parental line with the highest initial value, the ‘G × C’ population tended to higher I_AD_ values, with a uniform distribution ranging from 0.2 to 0.6. In contrast, the ‘B × C’ population showed a distribution concentrated between 0.2 and 0.4.

**Fig. 2:**
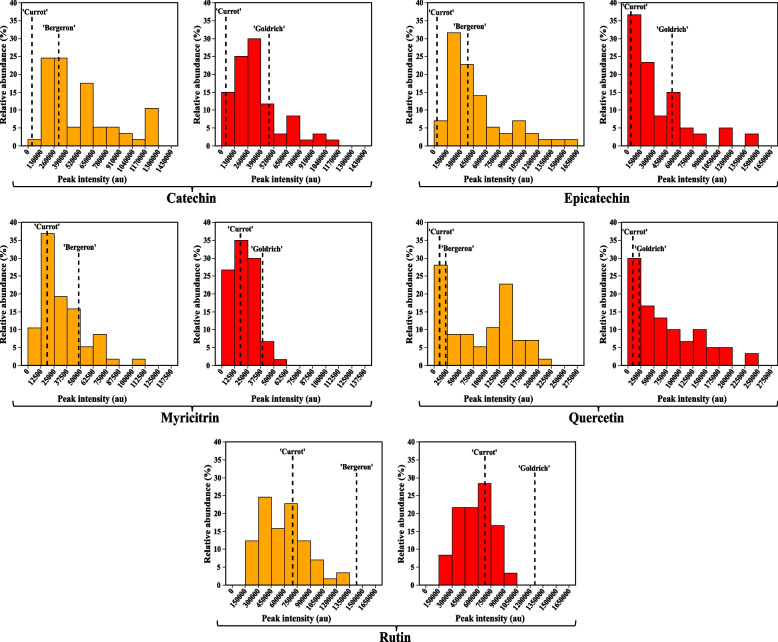
Frequency histograms of tentatively identified flavonoids (catechin, epicatechin, myricitrin, quercetin, and rutin), based on peak intensity in the ‘Bergeron’ × ‘Currot’ (orange bars) and ‘Goldrich’ × ‘Currot’ (red bars) populations

As for the flavonoid content, the distribution of peak intensities for catechin and epicatechin metabolites in both populations exhibited a similar pattern, with most genotypes clustering within the range defined by the parental values (Fig. 2). Notably, ‘Currot’ displayed a lower value compared to the maternal parents. However, a slight increase in catechin peak intensity was observed in the ‘B × C’ population, while in ‘G × C’, epicatechin exhibited two distinct regions: one centered around values near 150,000 and the other around 600,000. For myricitrin, the genotypes from the ‘B × C’ population displayed a broader dispersion of values, whereas ‘G × C’ exhibited a more compact distribution skewed toward the mid-to-low intensity range. In the case of quercetin, both populations showed higher peak intensity values compared to the parental lines, resulting in broader distributions. Finally, for rutin, lower peak intensity values compared to the parental genotypes predominated in both populations.

Correlations were analyzed using Pearson's correlation coefficient (r) to evaluate the relationships between fruit color parameters (I_AD_, SKC, FLSC, BLSC, %BLSC) and the content of secondary metabolites (catechin, epicatechin, myricitrin, quercetin, and rutin) for both populations (Table S1). With respect to the relationships among color parameters, SKC(L) was positively correlated with FLSC(L), with *r* = 0.44 (*P* < 0.01) in ‘B × C’ and *r* = 0.65 (*P* < 0.01) in ‘G × C’, as well as with SKC(h), with *r*≈0.45 (*P* < 0.01) for both populations. This moderate correlation between skin and flesh color suggests that fruits with lighter skin generally exhibit lighter flesh and a more uniform hue. Blush color parameters, specifically BLSC(L) and BLSC(h), were highly correlated (*r*≈0.95 (*P* < 0.01)) but negatively related to the % BLSC (*r*≈ − 0.80 (*P* < 0.01)) in both populations, indicating that fruits with higher blush lightness tend to have a smaller proportion of blush coverage.

Regarding flavonoid relationships, epicatechin exhibited positive correlations with catechin content, with *r* = 0.45 (*P* < 0.01) in ‘B × C’ and *r* = 0.27 (*P* < 0.05) in ‘G × C’. Additionally, rutin was strongly correlated with myricitrin (*r*≈0.84 (*P* < 0.01)) and moderately correlated with catechin, epicatechin, and quercetin (*r*≈0.4 (*P* < 0.01)) across all comparisons, with all interactions being positive.

In the analysis of relationships between color parameters and secondary metabolites, catechin showed weak negative correlations with BLSC(L) (*r* = − 0.29 (*P* < 0.05)) and BLSC(h) (*r* = − 0.25 (*P* < 0.05)) in the ‘B × C’ population, suggesting that fruits with a darker and more intense red blush tended to have lower catechin content. However, this trend was not consistent in ‘G × C’. In the ‘G × C’ population, catechin exhibited a significant correlation with I_AD_ (*r* = 0.37 (*P* < 0.01)), indicating that fruits with higher levels of chlorophyll may also have elevated catechin content. Myricitrin was negatively correlated with BLSC(h) (*r*≈ − 0.29 (*P* < 0.05)) and positively correlated with % BLSC (*r*≈0.35 (*P* < 0.01)) in both populations. This suggests that fruits with a redder blush and a higher proportion of blush coverage tend to have greater myricitrin content.

### Marker–trait associations for fruit color and flavonoids

**Fig. 3:**
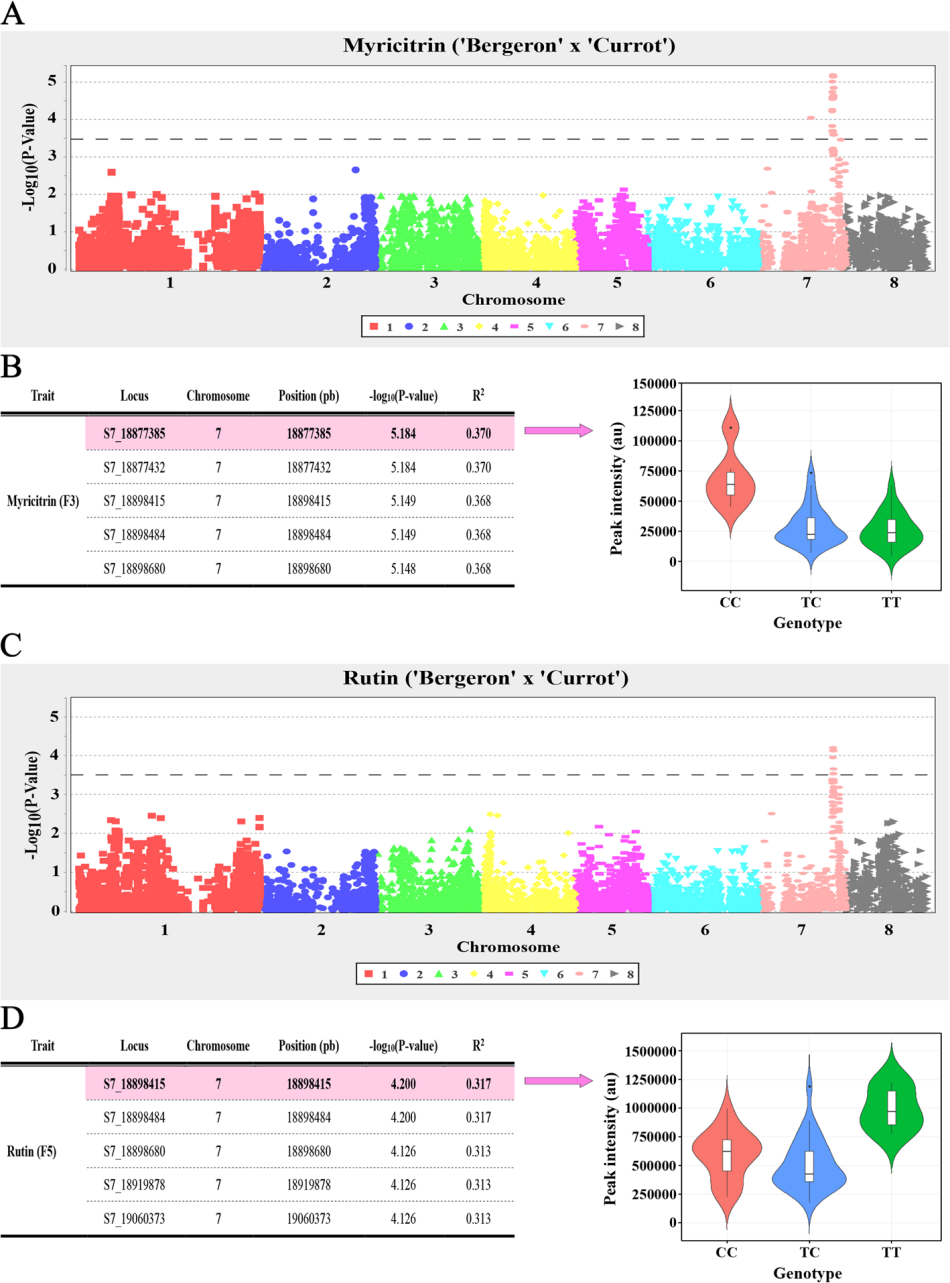
Manhattan plots for myricitrin and rutin in ‘Bergeron’ × ‘Currot’ population. The top panels display the -log_10_(*P*-value) distribution across the genome for Myricitrin (**A**) and Rutin (**C**). The middle panels summarize significant loci, their positions (bp), -log_10_(*P*-values), and R.^2^ values. The right panels show violin plots of peak intensity (au) for each genotype (CC, TC, TT) at the most significant loci for myricitrin (**B**) and rutin (**D**)

Marker-trait association was conducted on all metabolites using a previously published SNP data set from the ‘B × C’ and ‘G × C’ populations [22]. The most significant associations were identified for myricitrin, rutin and epicatechin (Table S2). The Manhattan plot of myricitrin and rutin in the ‘B × C’ population highlights significant associations primarily localized on chromosome 7 (Fig. 3A and C), specifically a narrow cluster of SNPs at the distal end of chromosome 7, around 19,000,000 bp. These SNPs display -log_10_(*P*-value) values of approximately 5 and 4 for myricitrin and rutin, respectively. In contrast, the remaining chromosomes exhibit a lower density of significant SNPs, characterized by more moderate and dispersed associations.

For myricitrin, locus S7_18877385 showed the strongest association, with a -log_10_(P-value) of 5.184 and an R^2^ value of 0.370, explaining a notable proportion of the phenotypic variation. Furthermore, the violin plot to the right illustrated that CC genotype showed greater variability and a higher median peak intensity compared to the CT and TT genotypes, suggesting a potential genotypic influence on the metabolite levels (Fig. 3B). For rutin, locus S7_18898415 emerged as the most significant, with a -log_10_(*P*-value) of 4.200 and an R^2^ value of 0.317, accounting for a substantial proportion of phenotypic variation. The violin plot revealed that the TT genotype exhibited the highest peak intensity (∼1,000,000 au) compared to the CC and TC genotypes (∼700,000 au and ∼500,000 au, respectively) (Fig. 3D).

**Fig. 4:**
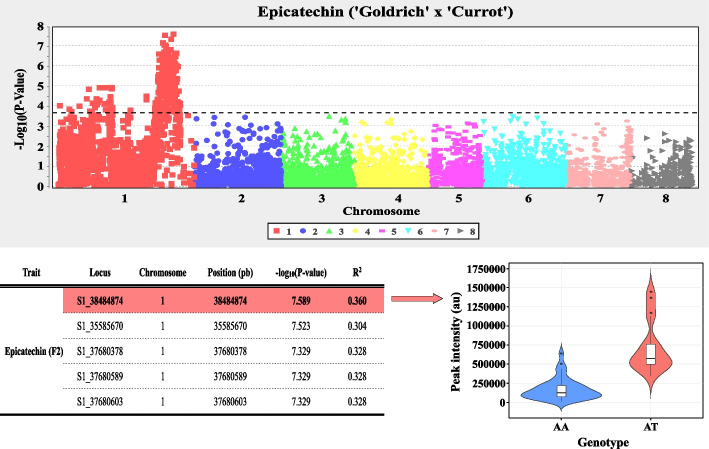
Manhattan plot for epicatechin in ‘Goldrich’ × ‘Currot’ population. The top panel displays the -log_10_*(P*-value) distribution across the genome. The middle panel summarizes significant loci, their positions (bp), -log_10_(*P*-values), and R^2^ values. The right panel shows violin plots of peak intensity (au) for each genotype (AA, AT) at the most significant loci

In the case of epicatechin in the ‘G × C’ population, the Manhattan plot revealed significant associations concentrated on chromosome 1 (Fig. 4). A prominent peak was observed at the terminal region of the chromosome, where multiple SNPs exceeded the significance threshold, with -log_10_(P-value) values nearing 7.5. This indicates a robust association with the epicatechin metabolite. The most significant locus, S1_38484874, located at 38,484,874 bp on chromosome 1, exhibited a -log_10_(*P*-value) of 7.589 and an R^2^ value of 0.360, explaining a considerable fraction of phenotypic variation. Loci located within the 35.0–38.0 Mb interval on chromosome 1, including S1_35585670, S1_37680378, S1_37680589, and S1_37680603, displayed similar -log_10_(P-value) and R^2^ values, reinforcing the importance of this chromosomal region for epicatechin accumulation. In contrast, other chromosomes displayed a lower density of significant SNPs, with moderate and scattered associations that did not match the levels of significance observed on chromosome 1. The violin plot showed that AT genotype displayed greater variability and a higher median peak intensity (∼500,000 au) compared to the AA genotype (∼125,000 au), suggesting a potential genotypic effect on epicatechin levels.

### QTL mapping for fruit color and flavonoids

QTL intervals associated with color-related and metabolomic traits were subsequently integrated into the genetic maps of ‘Bergeron’ (Figure S1), ‘Currot’ from the ‘B × C’ population (Figure S2), ‘Goldrich’ (Figure S3), and ‘Currot’ from the ‘G × C’ population (Figure S4). The most significant QTLs were identified and are presented below, selected based on a permutation test-derived LOD score threshold of 4.5.

**Fig. 5:**
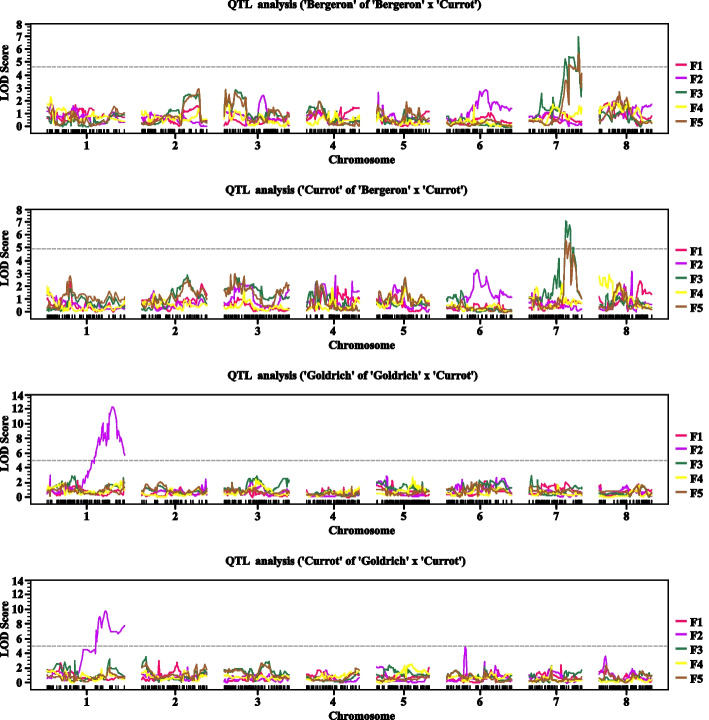
QTL analysis for each parent in ‘Bergeron’ × ‘Currot’ and ‘Goldrich’ × ‘Currot’ across the entire genetic map for flavonoid traits (catechin (F1), epicatechin (F2), myricitrin (F3), quercetin (F4), and rutin (F5))

In the ‘B × C’ population, a significant QTL for BLSC(L) was identified in ‘Bergeron’ parent on chromosome 4 at position 60.052 cM, corresponding to the locus S4_23098293 (< hkxhk >) (Table 2). This QTL accounted for 20.7% of the phenotypic variation, supported by a LOD score of 5.1 and a Kruskal–Wallis (K) statistic of 17.4, highlighting a strong association. In the previous marker–trait association analysis, this locus was not identified because all SNPs from both parents were included. For this reason, QTL analysis should be performed separately for each parent, as expected under the pseudo-testcross mapping model used in outcrossing species. In this context, markers such as < nnxnp > and < lmxll > segregate in a single heterozygous parent, enabling the detection of QTLs associated with alleles contributed specifically by that parent. Thus, parent-specific QTLs reflect the underlying segregation pattern rather than an increase in statistical significance. Furthermore, on chromosome 7, at position 46.460 cM, the locus S7_18877432 (< hkxhk >) was significantly associated with myricitrin concentration, explaining 40% of the variation with a LOD score of 6.3. Interestingly, the nearest locus (S7_18720617) was linked to rutin concentration, with a LOD score of 4.7, accounting for 31.5% of the phenotypic variation (Fig. 5). The QTLs related to these flavonols were supported by the non-parametric Kruskal–Wallis’s test (K values obtained were 13.7 and 13.6 respectively).

**Table 2:** Significant QTLs of traits related to color and flavonoids in the ‘Bergeron’ × ‘Currot’ population

**Parent**	**Trait**	**LG**^**a**^	**Position (cM)**	**Locus**	**Segregation**^**b**^	**LOD**	**K**	**%Expl.**^**c**^
‘Bergeron’	BLSC(L)	4	60.052	S4_23098293	< hkxhk >	5.1	17.4	20.7
	Myricitrin	7	46.460	S7_18877432	< hkxhk >	6.3	13.7	40.0
	Rutin	7	46.459	S7_18720617	< hkxhk >	4.7	13.6	31.5
‘Currot’	SKC(L)	5	67.547	S5_16344024	< hkxhk >	4.8	12.8	19.9
	I_AD_	5	18.166	S5_3625558	< nnxnp >	5.5	20.5	22.2
	Myricitrin	7	46.626	S7_18877385	< hkxhk >	6.5	13.7	40.6

^a^”LG” indicates the linkage group corresponding to the genetic map

^b^SNPs with < hkxhk > segregation are common to both parents, whereas < nnxnp > markers are present only in the male parent and < lmxll > markers only in the female parent

^c^"%Expl." represents the percentage of trait variation explained by the marker

In ‘Currot’, a QTL associated with SKC(L) was detected on chromosome 5 at position 67.547 cM, corresponding to the locus S5_16344024 (< hkxhk >). This QTL explained 19.9% of the phenotypic variation and had a LOD score of 4.8. Additionally, a significant QTL for I_AD_ was identified on chromosome 5 at position 18.166 cM (S5_3625558, < nnxnp >), accounting for 22.2% of the phenotypic variation with a LOD score of 5.5. Regarding flavonol concentrations, the locus S7_18877385 (< hkxhk >) on chromosome 7 at position 46.626 cM was strongly associated with myricitrin, explaining 40.6% of the phenotypic variation with a LOD score of 6.5. Notably, all significant QTLs identified for ‘Currot’ in the ‘B × C’ population showed consistent associations, with K values exceeding 13, underscoring the robustness of the genomic regions linked to these traits.

Major skin and flesh color QTLs linked to ‘G × C’ populations were previously identified at the end of chromosome 3. These traits are linked to the MYB genes family and other transcription factors, which explains a large proportion of the phenotypic variance in yellow to orange skin color [22]. In this study, new phenotypic data confirm these QTLs (Table 3). Additionally, new QTL analysis was carried out including new skin color and antioxidant related traits such as flavonoids. Thus, in the ‘G × C’ population, a significant QTL for SKC(L) was detected on chromosome 3 of ‘Goldrich’ parent at position 68.462 cM, corresponding to the locus S3_21873672 (< lmxll >) (Table 3). This locus had a LOD score of 6.4, a K statistic of 30, and explained 25.1% of the phenotypic variation. Similarly, for SKC(h), a QTL was identified on chromosome 3 at position 70.722 cM (locus S3_23316349, < lmxll >), with a much higher LOD score of 21.7, a K value of 63.1, and explaining 62.4% of the phenotypic variance. Regarding FLSC(L), a QTL on chromosome 1 at position 88.221 cM (locus S1_38997919, < lmxll >) was detected, with a LOD score of 5.4, a K value of 16.8, and a contribution of 21.7% to phenotypic explanation variance. For FLSC(h), a significant QTL co-localized with SKC(L) on chromosome 3 at position 68.462 cM (locus S3_21873672, < lmxll >), displaying a LOD score of 15.9, a K value of 53.5, and explaining 51.1% of the variation. Additionally, a QTL for I_AD_ was also identified on chromosome 5 at position 11.152 cM (locus S5_3774908, < hkxhk >), with a significant LOD score of 5.4, a K value of 16.4, and explaining 21.5% of the phenotypic variance. Lastly, the metabolomic trait epicatechin exhibited a QTL on chromosome 1 at position 79.676 cM (locus S1_35631447, < lmxll >), with a strong LOD score of 10.8, a K value of 30.2, and a substantial phenotypic explanation variance of 56.4% (Fig. 5).

**Table 3:** Significant QTLs of traits related to color and flavonoids in the ‘Goldrich’ × ‘Currot’ population

**Parent**	**Trait**	**LG**^**a**^	**Position (cM)**	**Locus**	**Segregation**^**b**^	**LOD**	**K**	**%Expl.**^**c**^
‘Goldrich’	SKC(L)	3	68.462	S3_21873672	< lmxll >	6.4	30.0	25.1
	SKC(h)	3	70.722	S3_23316349	< lmxll >	21.7	63.1	62.4
	FLSC(L)	1	88.221	S1_38997919	< lmxll >	5.4	16.8	21.7
	FLSC(h)	3	68.462	S3_21873672	< lmxll >	15.9	53.5	51.1
	I_AD_	5	11.152	S5_3774908	< hkxhk >	5.4	16.4	21.5
	Epicatechin	1	79.676	S1_35631447	< lmxll >	10.8	30.2	56.4
‘Currot’	SKC(L)	1	111.398	S1_40328756	< hkxhk >	5.0	19.1	20.3
	SKC(h)	3	72.859	S3_22658175	< hkxhk >	16.9	37.6	53.3
	FLSC(L)	1	111.398	S1_40328756	< hkxhk >	5.9	17.1	23.3
	FLSC(h)	3	72.859	S3_22658175	< hkxhk >	13.0	29.5	44.5
	Epicatechin	1	82.505	S1_33122023	< hkxhk >	8.9	20.2	49.6

^a^”LG” indicates the linkage group corresponding to the genetic map

^b^SNPs with < hkxhk > segregation are common to both parents, whereas < nnxnp > markers are present only in the male parent and < lmxll > markers only in the female parent

^c^"%Expl." represents the percentage of trait variation explained by the marker

On the other hand, in the ‘Currot’ parent, a QTL for SKC(L) was detected on chromosome 1 at position 111.398 cM (locus S1_40328756, < hkxhk >), with a LOD score of 5.0, a K value of 19.1, and explaining 20.3% of the phenotypic variance. For SKC(h), a significant QTL was located on chromosome 3 at position 72.859 cM (locus S3_22658175, < hkxhk >), with a LOD score of 16.9, a K value of 37.6, and explaining 53.3% of the variation. In terms of FLSC(L) a QTL was co-located with SKC(L) on chromosome 1 at position 111.398 cM, with a LOD score of 5.9, a K value of 17.1, and explaining 23.3% of the phenotypic variance. For FLSC(h), a QTL on chromosome 3 at position 72.859 cM (locus S3_22658175, < hkxhk >) showed a LOD score of 13.0, a K value of 29.5, and explained 44.5% of the variation. The fruit skin color QTLs identified in ‘Currot’ were nearly colocalized with those in ‘Goldrich’, as several common SNPs were mapped in both parental maps, indicating that they correspond to the same loci. Finally, for the metabolomic trait epicatechin, a QTL was detected on chromosome 1 at position 82.505 cM (locus S1_33122023, < hkxhk >), with a LOD score of 8.9, a K value of 20.2, and explaining 49.6% of the phenotypic variation.

### Phenolic acids and glycosides

**Fig. 6:**
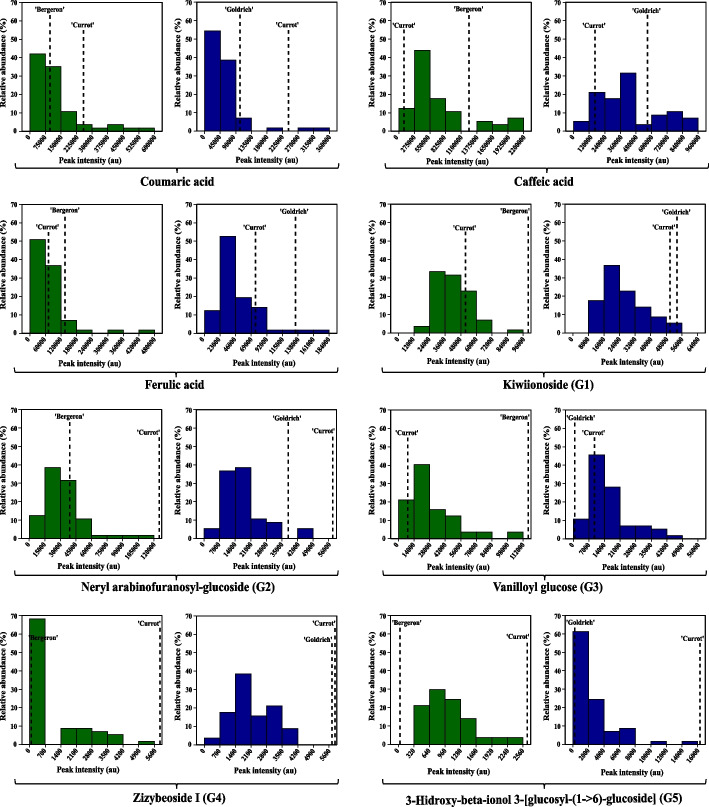
Frequency histograms of tentatively identified phenolic acids and glycosides (coumaric acid, caffeic acid, ferulic acid, kiwiionoside (G1), neryl arabinofuranosyl-glucoside (G2), vanilloyl glucose (G3), zizybeoside I (G4), and 3-hydroxy-beta-ionol 3-[glucosyl-(1- > 6)-glucoside] (G5)), based on peak intensity in the ‘Bergeron’ × ‘Currot’ (orange bars) and ‘Goldrich’ × ‘Currot’ (red bars) populations

For the frequency histograms of phenolic acids and glycosides, tentatively identified through untargeted UPLC-QToF-MS/MS analysis, peak intensities were utilized (Fig. 6). The distribution of coumaric acid and ferulic acid in both populations exhibits a similar pattern, where most seedlings display peak intensities lower than those of the parental lines. In contrast, caffeic acid tends to follow a bimodal distribution flanked by parental values, with a few individuals surpassing the peak intensities of the rest.

Regarding the glycosides, the distribution of peak intensities for kiwiionoside (G1) and neryl arabinofuranosyl-glucoside (G2) reveals a similar behavior, with individuals in the populations showing intensity levels lower than those of the parental lines, although the distribution remains normal. Conversely, in the ‘G × C’ population, the intensities for vanilloyl glucose (G3) and zizybeoside I (G4) shift to levels higher than those observed in the parental lines. Lastly, in the ‘B × C’ population, the distributions of 3-hydroxy-beta-ionol 3-[glucosyl-(1- > 6)-glucoside] (G5) and vanilloyl glucose (G3) and zizybeoside I (G4) highlight significant parental dispersion, with seedling intensities flanked by parental values. Notably, G5 in the ‘G × C’ population and G4 in the ‘B × C’ population exhibit a concentration of individuals at the lower intensity limit defined by the ‘Goldrich’ and ‘Bergeron’ parental lines, respectively. Thus, similar to the previous flavonoids quantified, some compounds displayed a positively skewed pattern with higher frequencies concentrated on the left side of the histogram, which is linked to the low frequency of alleles responsible for these traits. The relationships among phenolic acids (coumaric acid, caffeic acid, and ferulic acid) and glycosides (G1 to G5) in two apricot populations, ‘B × C’ and ‘G × C’, were analyzed using Pearson correlation coefficients (Table S3). Phenolic acids showed strong positive correlations in the ‘G × C’ population and moderate correlations in the ‘B × C’ population, particularly between coumaric acid and caffeic acid (0.73 (*P* < 0.01) in ‘G × C’ and 0.40 (*P* < 0.01) in ‘B × C’), as well as between caffeic acid and ferulic acid (0.69 (*P* < 0.01) in ‘G × C’ and 0.44 (*P* < 0.01) in ‘B × C’). Among phenolic acids and glycosides, kiwiionoside (G1) showed a negative correlation with caffeic acid (−0.26 (P < 0.05) in ‘B × C’ and −0.25 (*P* < 0.05) in ‘G × C’). Within the glycosides, vanilloyl glucose (G3) demonstrated a significant positive correlation with neryl arabinofuranosyl-glucoside (G2) (0.37 (*P* < 0.01) in ‘B × C’ and 0.31 (*P* < 0.01) in ‘G × C’), while zizybeoside I (G4) showed a negative correlation with kiwiionoside (G1) (−0.40 (*P* < 0.01) in ‘B × C’ and −0.38 (*P* < 0.01) in ‘G × C’). Additionally, 3-hydroxy-beta-ionol 3-[glucosyl-(1- > 6)-glucoside] (G5) displayed a moderate positive correlation with vanilloyl glucose (G3) (0.29 (*P* < 0.05) in ‘G × C’). These results highlighted significant interconnections between phenolic acids and glycosides.

### Marker–trait associations for phenolic acids and glycosides

Marker-trait association of the tentatively identified metabolites was conducted to provide a comprehensive overview of the loci associated with these phenolic acids and glycosides (Table S4). The Manhattan plot of neryl arabinofuranosyl-glucoside in the ‘B × C’ population highlights significant associations primarily located on chromosome 4 (Figure S5). In contrast, the remaining chromosomes display a lower density of significant SNPs, characterized by more moderate and scattered associations. For zizybeoside I, SNP associations were observed across the entire genome in the ‘B × C’ population, while in the ‘G × C’ population, associations were distributed across multiple chromosomes, with chromosomes 2 and 3 standing out as particularly significant.

**Fig. 7:**
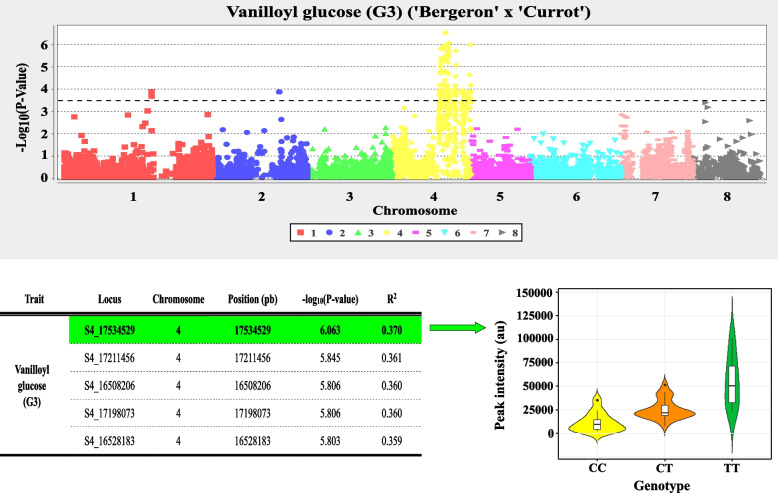
Manhattan plot for vanilloyl glucose (G3) in ‘Bergeron’ × ‘Currot’ population. The top panel displays the -log_10_(P-value) distribution across the genome. The middle panel summarizes significant loci, their positions (bp), -log_10_(P-values), and R^2^ values. The right panel shows violin plots of peak intensity (au) for each genotype (CC, CT, TT) at the most significant loci

For vanilloyl glucose in the ‘B × C’ population, the Manhattan plot (Fig. 7) revealed significant associations concentrated on chromosome 4. A notable peak was detected in the distal region of the chromosome, with multiple SNPs surpassing the significance threshold and exhibiting -log_10_(*P*-value) values close to 6. Among these, the most significant locus, S4_17534529 (positioned at 17,534,529 bp on chromosome 4), showed a -log_10_(*P*-value) of 6.063 and an R^2^ value of 0.370, explaining a substantial portion of the phenotypic variation. Additional loci located within approximately a 1 Mb window surrounding the most significant marker, such as S4_17211456, S4_16508206, S4_17198073, and S4_16528183, also demonstrated strong associations, with similar -log_10_(*P*-value) and R^2^ values, underscoring the critical role of this chromosomal region in vanilloyl glucose biosynthesis. In comparison, other chromosomes displayed a lower density of significant SNPs, with only scattered and moderate associations that did not approach the levels observed on chromosome 4. The violin plot on the right showed that the TT genotype exhibited the highest variability and a median peak intensity of approximately 50,000 au, substantially greater than the CT and CC genotypes, which showed median intensities of about 25,000 au and 12,500 au, respectively. This suggests a potential genotypic effect on vanilloyl glucose accumulation, with the TT genotype being associated with elevated metabolite levels. No significant associations were found regarding phenolic acids evaluated.

### QTL mapping for phenolic acid and glycosides

QTL regions linked to metabolomic traits were subsequently incorporated into the genetic maps of the parental lines, including ‘Bergeron’ and ‘Currot’ from the ‘B × C’ population, as well as ‘Goldrich’ and ‘Currot’ from the ‘G × C’ population (Figure S6). The most significant QTLs were determined based on a permutation test, with a threshold LOD score of 4.5, and are summarized below.

In the ‘Bergeron’ parental line from ‘B × C’, a significant QTL for vanilloyl glucose was mapped to chromosome 4 at 57.148 cM, associated with the locus S4_17799005 (< lmxll >) (Table 4). This QTL accounted for 49.2% of the phenotypic variation, supported by a robust LOD score of 8.4 and a K statistic of 19.1. Another QTL on chromosome 5 (S5_11251644) was strongly associated with zizybeoside I, with a LOD score of 11.0 and explaining 58.8% of the phenotypic variance.

**Table 4:** Significant QTLs for tentatively identified phenolic acids and glycosides in the ‘Bergeron’ × ‘Currot’ and ‘Goldrich’ × ‘Currot’ populations

**Parent**	**Trait**^**a**^	**LG**^**b**^	**Position (cM)**	**Locus**	**Segregation**^**c**^	**LOD**	**K**	**%Expl.**^**d**^
‘Bergeron’ (‘B × C’)	G3	4	57.148	S4_17799005	< lmxll >	8.4	19.1	49.2
	G4	5	35.918	S5_11251644	< lmxll >	11.0	41.3	58.8
‘Currot’ (‘B × C’)	G3	4	58.528	S4_16316561	< hkxhk >	7.6	22.2	45.9
	G4	5	49.569	S5_12526314	< hkxhk >	11.1	18.5	59.1
‘Goldrich’ (‘G × C’)	G4	2	29.682	S2_12310044	< hkxhk >	5.0	19.9	32.0
	G4	3	9.781	S3_1740069	< lmxll >	5.9	19.1	36.6
‘Currot’ (‘G × C’)	G4	2	39.064	S2_12868983	< hkxhk >	4.6	21.3	29.9

^a^Compound codes: vanilloyl glucose (G3) and zizybeoside I (G4)

^b^”LG” indicates the linkage group corresponding to the genetic map

^c^SNPs with < hkxhk > segregation are common to both parents, whereas < nnxnp > markers are present only in the male parent and < lmxll > markers only in the female parent

^d^"%Expl." represents the percentage of trait variation explained by the marker

In the ‘Currot’ parental line (‘B × C’), a QTL for vanilloyl glucose was detected on chromosome 4 at 58.528 cM, corresponding to the locus S4_16316561 (< hkxhk >). This QTL explained 45.9% of the phenotypic variation, with a LOD score of 7.6 and K values exceeding 20, emphasizing the reliability of this association. Furthermore, a QTL for zizybeoside I was identified on chromosome 5 at 49.569 cM, linked to the locus S5_12526314 (< hkxhk >), which accounted for 59.1% of the phenotypic variation and had a LOD score of 11.1. This QTL was previously identified through marker-trait association, confirming its high consistency.

In the ‘Goldrich’ parental line from ‘G × C’, a QTL associated with zizybeoside I was identified in two distinct regions. One was located on chromosome 2 at 29.682 cM, corresponding to the locus S2_12310044 (< hkxhk >), while the other was mapped to chromosome 3 at 9.781 cM (Table 4). Both loci exhibited LOD scores between 5 and 6, K values of 19–20, and explained 32–37% of the phenotypic variation.

In the ‘Currot’ parental line (‘G × C’), a QTL for zizybeoside I was detected on chromosome 2 at 39.064 cM, corresponding to the locus S2_12868983 (< hkxhk >). This QTL colocalized with a region previously identified in ‘Goldrich’, supporting the shared genetic basis. It displayed a LOD score of 4.6, a K value of 21.3, and explained 29.9% of the phenotypic variance. This QTL also showed consistency following marker-trait associations performed using the General Linear Model, as previously mentioned. Conversely, consistent with previous marker–trait associations, no significant associations were found for the phenolic acids evaluated.

### MQM mapping of key metabolites and candidate genes

**Fig. 8:**
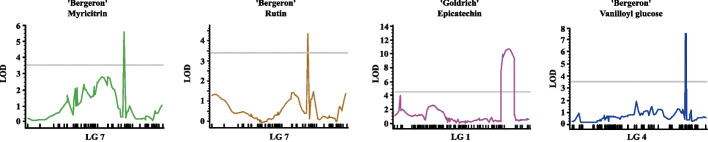
MQM analysis showing refined QTL profiles for the studied metabolites across linkage groups LG1, LG4, and LG7 in the ‘Bergeron’ × ‘Currot’ and ‘Goldrich’ × ‘Currot’ populations

MQM analysis was performed using the most significant SNP as a cofactor to refine QTL detection. This approach improved the resolution of the identified QTLs, confirming the major loci previously detected by interval mapping and reducing background noise (Fig. 8). The most prominent QTL signals were observed on linkage groups LG1, LG4, and LG7, showing consistent peaks. These results support the robustness of the detected associations and highlight key genomic regions controlling the studied metabolites.

Following QTL interval refinement, candidate gene analysis was performed by examining a 1 Mb genomic window surrounding the most significant markers. Specifically, regions from 18.5 to 19.5 Mb on LG7 for myricitrin and rutin, 37.5 to 38.5 Mb on LG1 for epicatechin, and 17.0 to 18.0 Mb on LG4 for vanilloyl glucose were explored to identify potential genes underlying these traits (Table S5). Regarding myricitrin and rutin, candidate genes within the QTL regions included key enzymes of the flavonoid biosynthetic pathway, such as chalcone isomerase (CAL8171271.1), cytochrome P450s (CAL8171086.1, CAL8171154.1, CAL8171197.1, CAL8171225.1, CAL8171243.1), and glycosyltransferases (CAL8171182.1), together with transcription factors from the MYB (CAL8171102.1, CAL8171122.1), bHLH (CAL8171278.1), and WD40 families (CAL8171168.1, CAL8171265.1), suggesting a role in flavonol accumulation.

Similarly, the refined QTL region for epicatechin revealed candidate genes involved in flavonoid biosynthesis and modification, including 2-oxoglutarate-dependent oxygenases (CAL8103297.1, CAL8103303.1, CAL8103309.1), cytochrome P450s (CAL8103459.1, CAL8103474.1), and an O-methyltransferase (CAL8103567.1), along with regulatory factors such as MYB (CAL8103331.1), NAC (CAL8103506.1), zinc finger (CAL8103085.1), and WD40 proteins (CAL8103318.1, CAL8103421.1).

In the case of vanilloyl glucose, candidate genes were mainly associated with glycosylation and secondary metabolism, including a glycosyltransferase (CAL8152635.1), an UDP-sugar-related gene (CAL8152566.1), cytochrome P450s (CAL8152413.1, CAL8152516.1), and several 2-oxoglutarate-dependent oxygenases (CAL8152525.1, CAL8152537.1, CAL8152539.1, CAL8152540.1, CAL8152554.1, CAL8152564.1). These were complemented by regulatory and transport-related genes, including MYB (CAL8152514.1), NAC (CAL8152473.1), WD40 (CAL8152561.1), and MATE transporters (CAL8152570.1, CAL8152592.1, CAL8152593.1), likely contributing to metabolite accumulation and transport.

## Discussion

### Biochemical basis of fruit color, antioxidant attributes, and taste-related traits: Roles of flavonoids, phenolic acids, and glycosides

In terms of fruit color, the intense orange skin and flesh color of apricot fruits is primarily attributed to the accumulation of carotenoids. Interestingly, the hue angle (hº) derived from the CIE Lab* color space has shown an exceptionally strong correlation—often exceeding 0.95—with total carotenoid content in various fruit species, including apricot. This makes hº a reliable, non-destructive proxy for estimating carotenoid concentration during breeding or postharvest evaluation [41]. On the other hand, the reddish coloration known as blush is primarily due to the accumulation of anthocyanins, particularly cyanidin-3-O-rutinoside [42]. Having said that, apricot undergoes a dynamic color transition from green to yellow, orange, or red, driven by chlorophyll degradation and the biosynthesis of pigments such as carotenoids and flavonoids like anthocyanins [43]. Together, these traits are the main contributors to the fruit's coloration.

In our study, frequency histograms occasionally revealed a normal distribution in both populations regarding color properties, highlighting the phenotypic diversity encompassed by the parental lines in terms of skin and flesh color parameters. However, a slight tendency toward a bimodal distribution in the skin color of ‘G × C’ can be observed, distinguishing two groups: one with more yellowish tones and another with more orange hues, which is likely associated with the presence of major genes. This underscores the critical role of the genetic material from each progenitor in determining fruit color in their offspring. Fruit blush color and its percentage are largely determined by parental genotypes; however, genotype × environment interactions also appear to play a role. For instance, a shift toward more intense red hues in fruit blush was observed, contrasting with the findings evaluated the same populations in 2012–2013 [44]. This suggests that projected environmental changes, particularly in temperature [45], may affect the expression and significance of quality-related traits, potentially altering QTL detection in bi-parental populations over different years. Moreover, the chlorophyll index (I_AD_) is sometimes used as a harvest criteria for fruit trees such as peach, but it’s important to note that this index varies depending on the genotype and even the fruit tree species [46]. For instance, while an I_AD_ value of around 0.8–1 may be suitable for commercially harvested peaches, in apricots this value tends to be closer to 0.5.

As previously mentioned, the anthocyanin cyanidin-3-O-rutinoside is strongly associated with red coloration and is therefore responsible for the characteristic red blush in *Prunus* spp. [47]. However, other less-studied flavonoid compounds also play an important role as natural antioxidants, offering health benefits [48]. In our study, the content of catechin, epicatechin, myricitrin, quercetin and rutin exhibited notably asymmetric distributions, particularly catechin, epicatechin, and myricitrin, which showed a positively skewed pattern. This type of skewed distribution may reflect polygenic inheritance combined with dominance effects toward lower metabolite accumulation.

Regarding the relationship between color traits and flavonoids, no strong correlations were found in this study, aligning with previous reports that observed no significant correlation with flesh color in apricots [49]. Nonetheless, studies have reported significant positive correlations between blush color and the presence of anthocyanins [50], a group of phenolic compounds responsible for stable pigmentation in many plant species such as apple and plum, where they occur as glycosylated derivatives. These anthocyanins are regulated by MYB family genes, which are known to play a pivotal role in modulating metabolic pathways related to the biosynthesis of flavonoids and other secondary compounds [51].

In our research, we successfully identified flavonoids grouped into the subclasses of flavonols and flavan-3-ols. These compounds have been widely proposed as beneficial for human health in numerous studies, demonstrating potential protective effects against oxidative stress, as well as neuroprotective, antidiabetic, anticancer, and antimicrobial properties [52]. For flavonols, their biosynthesis is regulated by a series of key enzymes within the phenylpropanoid pathway, where flavonol synthase (FLS) plays a critical role. Conversely, the biosynthesis of flavan-3-ol monomers ((+)-catechin and (-)-epicatechin) begins with 4-coumaroyl-CoA, a product of the phenylpropanoid pathway, which serves as a precursor in the flavonoid biosynthetic pathway. Enzymes such as chalcone synthase (CHS), chalcone isomerase (CHI), and flavonoid 3-hydroxylase (F3H) are essential for the production of dihydroflavonols, the substrates for both flavan-3-ols and anthocyanins. Dihydroflavonol-4-reductase (DFR) catalyzes the formation of leucoanthocyanidins, which serve as substrates for leucoanthocyanidin reductase (LAR) to produce (+)-catechin, and for anthocyanidin synthase (ANS) to produce anthocyanidins. These anthocyanidins are subsequently utilized by anthocyanidin reductase (ANR) to produce (-)-epicatechin [53]. These interconnections within the biosynthetic pathways are reflected in the significant correlations observed in our research.

Regarding organic acids and glycosides, apricots contain a rich profile that significantly influences their taste and nutritional value. Malic and citric acid are the most prominent, contributing to the fruit’s characteristic tartness and playing essential roles in cellular metabolism [54]. In terms of glycosides, quercetin glucoside, not only supports the fruit’s health-promoting properties but also participates in the release of aromatic and bioactive molecules during ripening, subtly shaping its flavor profile [55]. However, other less-studied glycosides and phenolic acids also play an important role in taste and flavor showing significant differences among the evaluated populations. (1) Phenolic acids: coumaric, caffeic, and ferulic acids; (2) glycosides: kiwiionoside, neryl arabinofuranosyl-glucoside, vanilloyl glucose, zizybeoside I, and 3-hydroxy-beta-ionol 3-[glucosyl-(1- > 6)-glucoside]. Similar to the previously quantified flavonoids, some phenolic acids and glycosides exhibited a positively skewed distribution, with higher frequencies concentrated on the left side of the histogram, likely reflecting polygenic inheritance combined with dominance effects favoring lower metabolite accumulation. On the other hand, positives correlations among the tentatively identified phenolic acids, namely coumaric, caffeic, and ferulic acids, were found suggesting shared biosynthetic pathways (Figure S7). These compounds serve as intermediates or end-products of the phenylpropanoid pathway, a central metabolic route in plants that contributes to the synthesis of various phenolic compounds involved in plant defense, signaling, dormancy release [56] in apricot and peach and structural integrity [57–59].

Regarding glycosides compounds, these belong to the subclass of glycosylated metabolites, which play a crucial role in the storage, transport, and detoxification of secondary metabolites [60]. Notably, vanilloyl glucose (G3) is a phenylpropanoid derivative, while kiwiionoside (G1), neryl arabinofuranosyl-glucoside (G2), zizybeoside I (G4), and 3-hydroxy-beta-ionol 3-[glucosyl-(1- > 6)-glucoside] (G5) are derived from terpenoid metabolism. The presence of these glycosides highlights the intricate metabolic interactions in plants, particularly between the phenylpropanoid and terpenoid biosynthetic pathways, both of which contribute to fruit quality, dormancy release [56] and plant defense [61]. Among phenolic acids and glycosides, low but significant correlations were found, suggesting a slight interconnection between the two. Differences in correlation patterns between the ‘B × C’ and ‘G × C’ populations likely reflect their distinct genetic backgrounds and allelic segregation, which influence metabolite accumulation and trait relationships. Moreover, although some correlations were weak such values are expected for complex traits controlled by multiple loci and may still indicate biologically relevant associations, although they should be interpreted with caution. Rather than performing a global correlation analysis including all detected metabolites, we focused on these biologically relevant groups to reduce noise derived from unrelated or low-abundance compounds and to facilitate a more interpretable and biologically meaningful analysis.

### Marker trait association and QTL identification

In this study, a SNP dataset from the ‘B × C’ and ‘G × C’ populations, along with antioxidant, taste, and flavor-related traits, was used to detect key marker–trait associations without distinguishing between parental lines. Subsequently, QTL mapping was performed, differentiating between female and male parents. In this context, different mapping approaches were applied to ensure robust QTL detection. Interval mapping provided an initial localization of QTLs, while the non-parametric Kruskal–Wallis test allowed the identification of marker–trait associations without assuming normal data distribution, which is particularly suitable for metabolite data. Furthermore, MQM analysis, performed for metabolites showing significant QTLs, improved mapping resolution by accounting for background genetic variation through the inclusion of cofactors. Overall, MQM refined QTL positions and strengthened the confidence in the detected associations compared to IM and Kruskal–Wallis approaches. Regarding color-related QTLs, it is noteworthy to highlight the highly significant loci for skin color (SKC(h)) and flesh color (FLSC(h)) identified in the ‘G × C’ population. These QTLs were located on chromosome 3, aligning with those previously described [22] and in other *Prunus* species, such as peach [62] and sweet cherry [63]. This highlights the importance of this genomic region as a target for further research into fruit coloration. In other species like plum, molecular markers have already been developed, particularly focusing on the MYB gene family, which plays a crucial role in determining skin color [64], and transposon-based markers for flesh color [65].

For the flavonol-related QTLs (myricitrin and rutin) identified at the distal end of chromosome 7, this region is of substantial interest for unraveling the genetic background regulating the biosynthesis of these flavonoids. While no QTLs for these specific flavonols have been reported within the *Prunus* genus, this region coincides with those described for total flavonoid content [26]. Additionally, in other species such as tomato, where rutin plays a pivotal role, and apple, QTLs have been identified for these compounds [66, 67].

Finally, the QTL for epicatechin deserves special attention due to its high significance and the critical role of this metabolite in flavonoid pathways, serving as a precursor of proanthocyanidins (PAs) [68]. PAs influence the bitter taste and astringency of fruits, vegetables, and plant-derived products, such as red wine and tea [69]. In plum, the *R2R3 MYB700* gene has been identified as an activator of epicatechin biosynthesis, marking it as a key factor potentially replicable in apricot [70]. The epicatechin QTL was mapped to the distal end of chromosome 1, consistent with findings in plum [71]. In plum, both catechin and epicatechin, due to their structural isomerism, were mapped to the same region through the separation of pulp and skin [71]. However, in our study, possibly due to noise in the collected data and the analysis of the edible portion (combining both pulp and skin), the catechin-related QTL was not detected.

As for organic acids, previous studies in *Prunus* species have identified significant QTLs. For instance, QTLs for malic and citric acid concentrations have been mapped in peach and apricot, demonstrating their association with fruit acidity and flavor profiles [23, 72]. Furthermore, research on QTLs for other quality traits, such as acidity and sweetness in *Prunus*, suggests potential interrelations with the biosynthetic pathways of coumaric, caffeic, and ferulic acids. However, these connections warrant further investigation to understand how these pathways may interact or share regulatory elements, especially since we found no significant associations through GLM or QTL analysis. The absence of detectable QTLs for these traits likely reflects complex genetic regulation and environmental interactions, necessitating larger population sizes or alternative mapping approaches to enhance resolution [73].

Regarding glycosides compounds, mapping QTLs revealed regions with potential regulatory roles in their biosynthesis and accumulation. For example, QTLs linked to vanilloyl glucose (G3) on chromosome 4 may overlap with loci controlling fruit ripening and developmental timing in apricot [74], indicating a potential relationship between these traits. Similarly, QTLs associated with terpenoid-derived glycosides, such as G2 (also located on chromosome 4), may be related to maturation processes. In the case of zizybeoside I (G4), a clear polygenic behavior has been observed, making its analysis challenging.

Research in other *Prunus* species has identified QTLs linked to glycosylated metabolites [75], which could provide a framework for interpreting the findings of this study, in addition to highlighting the importance of glycosylation [76]. However, this is the first time that a QTL associated with vanilloyl glucose has been identified; previous studies have only reported its health benefits [77]. The possible functional significance of these glycosides in fruit development and ripening, and particularly their contributions to sensory attributes and stress resilience, presents exciting opportunities for breeding programs aimed at improving fruit quality.

These findings underscore mainly the genetic control of flavonoid and glycoside biosynthesis in apricot, highlighting their roles as bioactive compounds with health-promoting properties and contributions to fruit quality. The integration of metabolomic, genomic, and QTL mapping approaches advances our understanding of the genetic basis of these metabolites and represents a crucial step toward the development of MAS strategies aimed at enhancing metabolite content and precision breeding for high-quality apricot varieties.

## Conclusions

This study has enabled us to identify, through untargeted UPLC-QToF-MS/MS analysis, the most significant compounds in two apricot populations (‘B × C’ and ‘G × C’), including flavonoids, phenolic acids, and glycosides present in apricot fruit.

In total, thirteen compounds were tentatively identified: five flavonoids (catechin, epicatechin, myricitrin, quercetin, and rutin), three phenolic acids (coumaric, caffeic, and ferulic acids), and five glycosides (kiwiionoside, neryl arabinofuranosyl-glucoside, vanilloyl glucose, zizybeoside I, and 3-hydroxy-beta-ionol 3-[glucosyl-(1 → 6)-glucoside]). The flavonoid-related results revealed the localization of major QTLs for epicatechin on linkage group 1, explaining 56.4% of the phenotypic variation and supported by a LOD score of 10.8 (K = 30.2). In addition, QTLs for myricitrin and rutin; two highly positively correlated compounds, were identified on linkage group 7, accounting for 40% and 31.5% of the phenotypic variation, respectively (LOD = 6.3 and 4.7; K = 13.7 and 13.6). These flavonoids are mainly associated with health-promoting properties due to their strong antioxidant capacity rather than with color-related traits. Regarding glycosides, a major QTL for vanilloyl glucose (G3) was detected on linkage group 4, explaining 49.2% of the phenotypic variation (LOD = 8.4; K = 19.1), while zizybeoside I (G4) a major QTL on linkage group 5 explaining approximately 58% of the phenotypic variation was identified, despite additional loci suggesting a partially polygenic architecture. Although no significant QTLs were detected for the phenolic acids (coumaric, caffeic, and ferulic acids), positive correlations among them reflected their interconnected biosynthetic pathways.

Together, these results demonstrate the genetic control of flavonoid and glycoside biosynthesis in apricot and represent a significant step toward the development of MAS strategies. Such strategies could facilitate the breeding of apricot varieties with improved metabolite content, thereby enhancing both fruit quality and functional properties. Ultimately, these findings reinforce the value of apricots as a functional food for health-conscious consumers and provide a foundation for future research on the genetic regulation of secondary metabolism in *Prunus* species.

## Supplementary Information

Supplementary Material 1: Table S1. Pearson correlation analysis of tentatively identified flavonoids and color traits.

Supplementary Material 2: Table S2. Summary table of trait-marker associations for tentatively identified flavonoids using TASSEL v5 in the ‘B × C’ and ‘G × C’ populations.

Supplementary Material 3: Table S3. Pearson correlation analysis of tentatively identified phenolic acids and glycosides.

Supplementary Material 4: Table S4. Summary table of trait-marker associations for tentatively identified phenolic acids and glycosides using TASSEL v5 in the ‘B × C’ and ‘G × C’ populations.

Supplementary Material 5: Table S5. Candidate genes associated with myricitrin, rutin, epicatechin, and vanilloyl glucose identified within QTL regions and functionally annotated using eggNOG-mapper.

Supplementary Material 6: Figure S1. QTL analysis for the ‘Bergeron’ parent in ‘B × C’ across the entire genetic map for color and flavonoid traits.

Supplementary Material 7: Figure S2. QTL analysis for the ‘Currot’ parent in ‘B × C’ across the entire genetic map for color and flavonoid traits.

Supplementary Material 8: Figure S3. QTL analysis for the ‘Goldrich’ parent in ‘G × C’ across the entire genetic map for color and flavonoid traits.

Supplementary Material 9: Figure S4. QTL analysis for the ‘Currot’ parent in ‘B × C’ across the entire genetic map for color and flavonoid traits.

Supplementary Material 10: Figure S5. Above the figure is the Manhattan plot for neryl arabinofuranosyl-glucoside (G2) in the ‘B × C’ population. Below are the Manhattan plots for zizybeoside I (G4) in both the ‘B × C’ and ‘G × C’ populations. The red ellipses highlight the most significant chromosomes in each of the Manhattan plots.

Supplementary Material 11: Figure S6. QTL analysis for each parent in ‘Bergeron’ × ‘Currot’ (‘B × C’) and ‘Goldrich’ × ‘Currot’ (‘G × C’) populations across the entire genetic map for tentatively identified phenolic acids and glycosides.

Supplementary Material 12: Figure S7. Biosynthetic pathway of vanilloyl glucose and its interconnection with the phenylpropanoid and anthocyanin pathways. Enzymes and intermediates are depicted to highlight the metabolic flow and regulatory nodes within these interconnected pathways.

## Data Availability

The data supporting the findings of this study, derived from marker–trait (genotype–phenotype) associations, are included in Supplementary Tables S2 and S4.

## Abbreviations

‘B × C’: Bergeron x Currot
‘G × C’: Goldrich x Currot
BLSC: Blush Color
CU: Chill Units
FLSC: Flesh Color
HPLC: High-Performance Liquid Chromatography
I_AD_: Index of Absorption of Chlorophyll
KEGG: Kyoto Encyclopedia of Genes and Genomes
LG: Linkage Group
LOD: Logarithm of the Odds
MAS: Marker Assisted Selection
MQM: Multiple QTL Mapping
QTL: Quantitative Trait Locus
SKC: Skin Color
SNP: Single Nucleotide Polymorphism
UPLC-QToF-MS/MS: Ultra Performance Liquid Chromatography Quadrupole Time-of-Flight Mass Spectrometry/Mass Spectrometry
Vis–NIR: Visible Near-Infrared
