# Supplementary material for: Identification of QTLs linked to bioactive flavonoids and glycosides in the apricot fruit (Prunus armeniaca L.)
Source: BMC Genomics. 2026 May 30;27:626. doi: 10.1186/s12864-026-12989-0 (PMC13386964; doi:10.1186/s12864-026-12989-0)
Supplement: Supplementary file 1 — Supplementary Material 1: Table S1. Pearson correlation analysis of tentatively identified flavonoids and color traits. [file 12864_2026_12989_MOESM1_ESM.docx]

**Table S1.** Pearson correlation analysis of tentatively identified flavonoids and color traits.

| **2023 (Year)^a^** | **I_AD_** | **SKC(L)** | **SKC(h)** | **FLSC(L)** | **FLSC(h)** | **BLSC(L)** | **BLSC(h)** | **%BLSC** | **F1** | **F2** | **F3** | **F4** | **F5** |
| --- | --- | --- | --- | --- | --- | --- | --- | --- | --- | --- | --- | --- | --- |
| **I_AD_ ^b^** | 1.00 | -0.03 | 0.02 | -0.01 | -0.07 | 0.14 | 0.19 | 0.00 | **0.37**** | 0.21 | -0.20 | -0.10 | -0.04 |
| **SKC(L)^b^** | **-0.28*** | 1.00 | **0.45**** | **0.65**** | **0.52**** | **0.40**** | **0.28*** | -0.16 | -0.05 | -0.12 | 0.09 | 0.05 | 0.11 |
| **SKC(h) ^b^** | **0.25*** | **0.44**** | 1.00 | 0.12 | **0.85**** | 0.09 | 0.19 | -0.16 | -0.11 | 0.09 | -0.03 | 0.03 | 0.02 |
| **FLSC(L) ^b^** | -0.14 | **0.44**** | -0.05 | 1.00 | **0.36**** | 0.17 | 0.03 | 0.09 | -0.09 | **-0.42**** | 0.05 | 0.07 | 0.03 |
| **FLSC(h) ^b^** | -0.17 | **0.30*** | **0.34**** | **0.41**** | 1.00 | 0.16 | 0.18 | -0.13 | -0.22 | -0.10 | -0.05 | 0.03 | -0.03 |
| **BLSC(L) ^b^** | -0.15 | **0.35**** | 0.10 | 0.06 | 0.13 | 1.00 | **0.95**** | **-0.75**** | 0.15 | -0.17 | -0.15 | 0.03 | -0.03 |
| **BLSC(h) ^b^** | 0.06 | 0.14 | 0.09 | -0.11 | -0.01 | **0.93**** | 1.00 | **-0.81**** | 0.13 | -0.14 | **-0.25*** | -0.04 | -0.12 |
| **%BLSC ^b^** | 0.08 | -0.24 | -0.08 | 0.09 | -0.02 | **-0.81**** | **-0.83**** | 1.00 | 0.00 | 0.05 | **0.36**** | 0.19 | 0.21 |
| **Catechin (F1)** | 0.14 | -0.15 | -0.10 | 0.10 | 0.15 | **-0.29*** | **-0.25*** | 0.17 | 1.00 | **0.27*** | 0.14 | 0.00 | **0.32**** |
| **Epicatechin (F2)** | 0.22 | 0.02 | -0.02 | 0.10 | 0.21 | 0.15 | 0.17 | -0.18 | **0.45**** | 1.00 | 0.09 | 0.11 | **0.28*** |
| **Myricitrin (F3)** | -0.08 | -0.01 | -0.02 | 0.17 | 0.14 | -0.21 | **-0.29*** | **0.35**** | **0.32**** | **0.26*** | 1.00 | **0.49**** | **0.86**** |
| **Quercetin (F4)** | -0.11 | 0.04 | -0.14 | 0.15 | 0.09 | 0.14 | 0.06 | -0.03 | 0.07 | 0.20 | **0.25*** | 1.00 | **0.45**** |
| **Rutin (F5)** | -0.01 | 0.12 | -0.02 | 0.24 | 0.11 | -0.08 | -0.15 | 0.16 | **0.49**** | **0.44**** | **0.84**** | **0.33**** | 1.00 |

^a^ The correlation is significant at the 0.05 (*) and 0.01 (**) levels. Below the diagonal, the results correspond to the ‘Bergeron’ × ‘Currot’ population, while those above the diagonal represent the correlation for the ‘Goldrich’ × ‘Currot’ population.

^b^ The meaning of the abbreviations: I_AD_: Index of chlorophyll degradation; SKC: Skin color; FLSC: Flesh color; BLSC: Blush color; %BLSC: Percentage of blush color.
