# Supplementary material for: Identification of QTLs linked to bioactive flavonoids and glycosides in the apricot fruit (Prunus armeniaca L.)
Source: BMC Genomics. 2026 May 30;27:626. doi: 10.1186/s12864-026-12989-0 (PMC13386964; doi:10.1186/s12864-026-12989-0)
Supplement: Supplementary file 3 — Supplementary Material 3: Table S3. Pearson correlation analysis of tentatively identified phenolic acids and glycosides. [file 12864_2026_12989_MOESM3_ESM.docx]

**Table S3.** Pearson correlation analysis of tentatively identified organic acids and glucosides.

| **2023 (Year)^a^** | **Coumaric acid** | **Caffeic acid** | **Ferulic acid** | **G1** | **G2** | **G3** | **G4** | **G5** |
| --- | --- | --- | --- | --- | --- | --- | --- | --- |
| **Coumaric acid** | 1.00 | **0.73**** | **0.67**** | -0.18 | 0.13 | 0.08 | 0.16 | -0.12 |
| **Caffeic acid** | **0.40**** | 1.00 | **0.69**** | **-0.25*** | 0.15 | 0.02 | **0.31**** | -0.08 |
| **Ferulic acid** | **0.42**** | **0.44**** | 1.00 | -0.15 | 0.14 | 0.02 | 0.18 | -0.11 |
| **Kiwiionoside (G1)** | **-0.26*** | **-0.26*** | -0.20 | 1.00 | 0.24 | 0.12 | **-0.38**** | 0.03 |
| **Neryl arabinofuranosyl-glucoside (G2)** | 0.10 | 0.10 | -0.13 | -0.10 | 1.00 | **0.31**** | 0.15 | 0.06 |
| **Vanilloyl glucose (G3)** | 0.21 | **0.27*** | 0.06 | -0.03 | **0.37**** | 1.00 | 0.13 | **0.29*** |
| **Zizybeoside I (G4)** | 0.00 | 0.12 | -0.08 | **-0.40**** | **0.43**** | 0.07 | 1.00 | 0.14 |
| **3-Hydroxy-beta-ionol 3-[glucosyl-(1->6)-glucoside] (G5)** | 0.19 | 0.03 | -0.01 | -0.18 | 0.10 | -0.07 | 0.12 | 1.00 |

^a^ The correlation is significant at the 0.05 (*) and 0.01 (**) levels. Below the diagonal, the results correspond to the ‘Bergeron’ × ‘Currot’ population, while those above the diagonal represent the correlation for the ‘Goldrich’ × ‘Currot’ population.
