# Supplementary figures and images for: Identification of QTLs linked to bioactive flavonoids and glycosides in the apricot fruit (Prunus armeniaca L.)
Source: BMC Genomics. 2026 May 30;27:626. doi: 10.1186/s12864-026-12989-0 (PMC13386964; doi:10.1186/s12864-026-12989-0)

QTL ANALYSIS ('BERGERON' OF 'BxC')

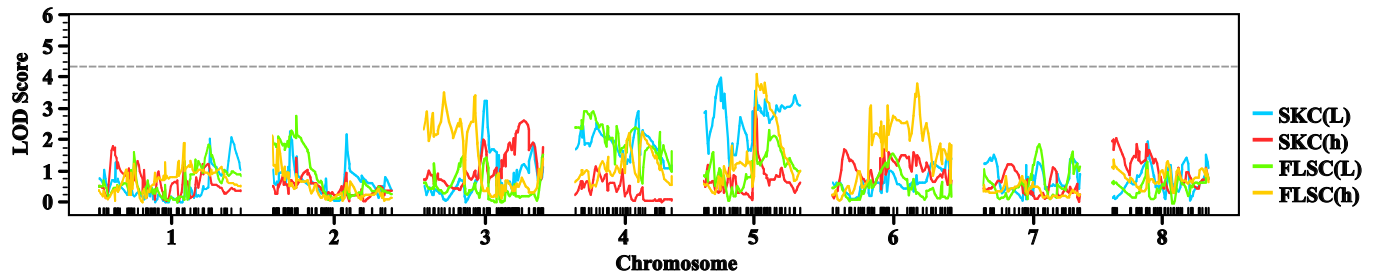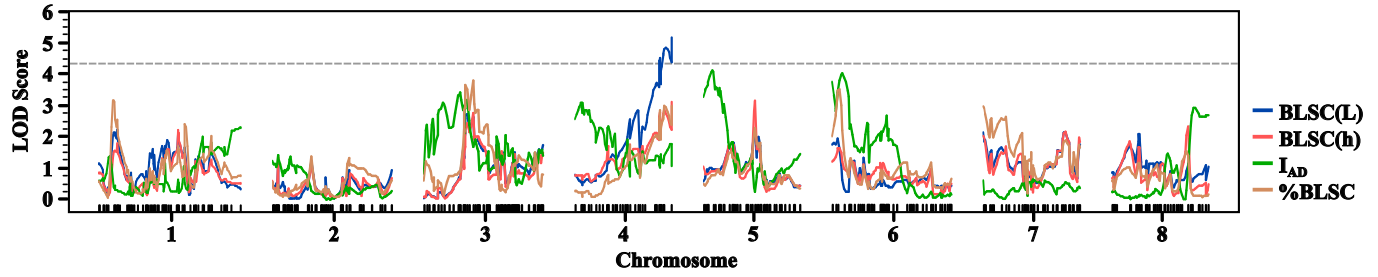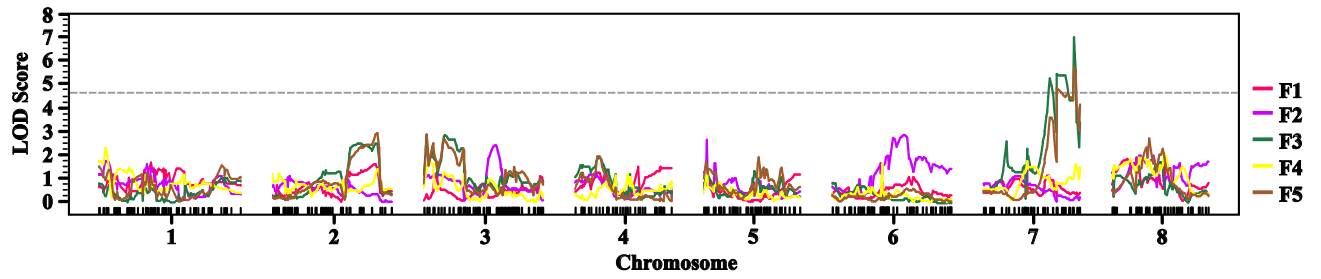

Supplement: Supplementary file 6 — Supplementary Material 6: Figure S1. QTL analysis for the ‘Bergeron’ parent in ‘B × C’ across the entire genetic map for color and flavonoid traits. [file 12864_2026_12989_MOESM6_ESM.pdf]

# QTL ANALYSIS ('CURROT' OF 'BxC')

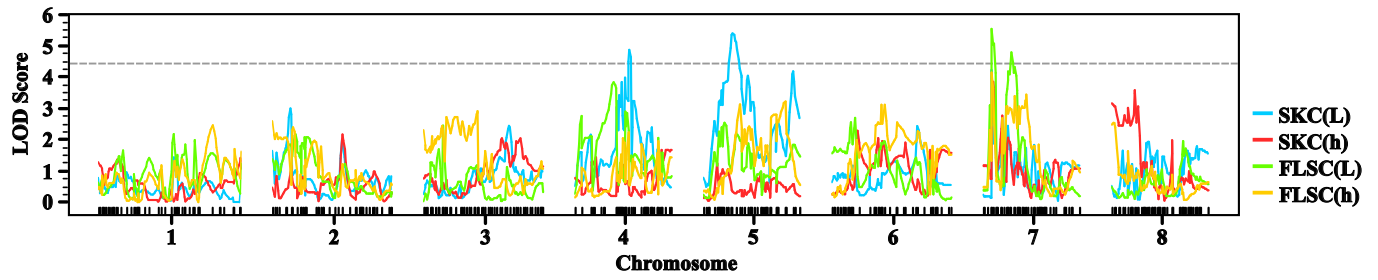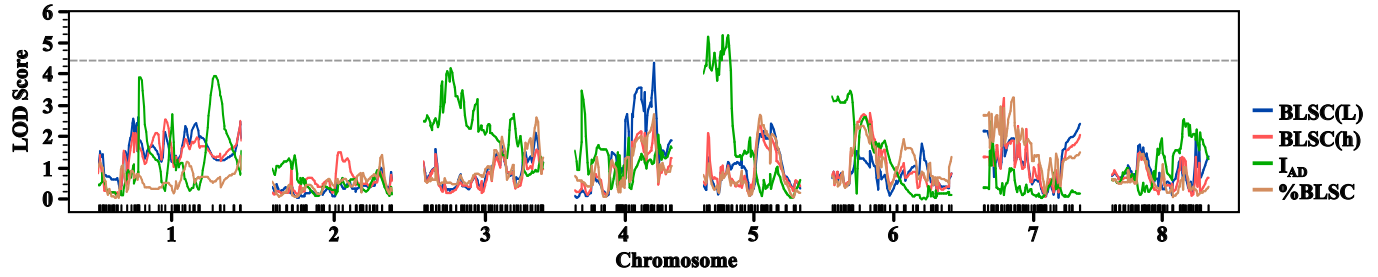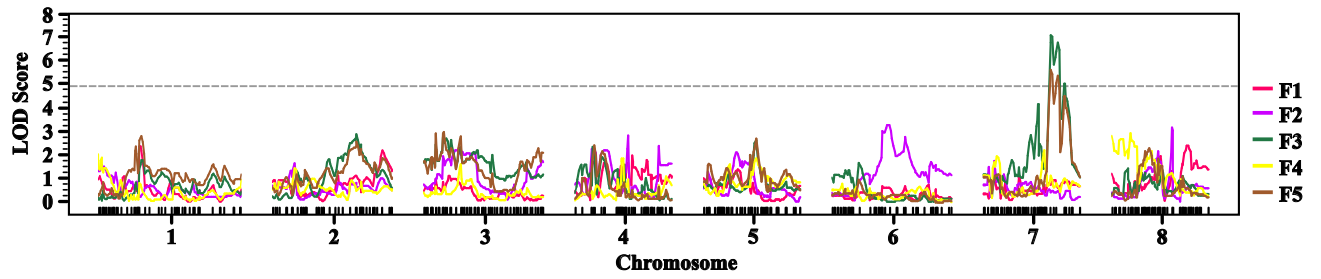

Supplement: Supplementary file 7 — Supplementary Material 7: Figure S2. QTL analysis for the ‘Currot’ parent in ‘B × C’ across the entire genetic map for color and flavonoid traits. [file 12864_2026_12989_MOESM7_ESM.pdf]

# QTL ANALYSIS ('GOLDRICH' OF 'GxC')

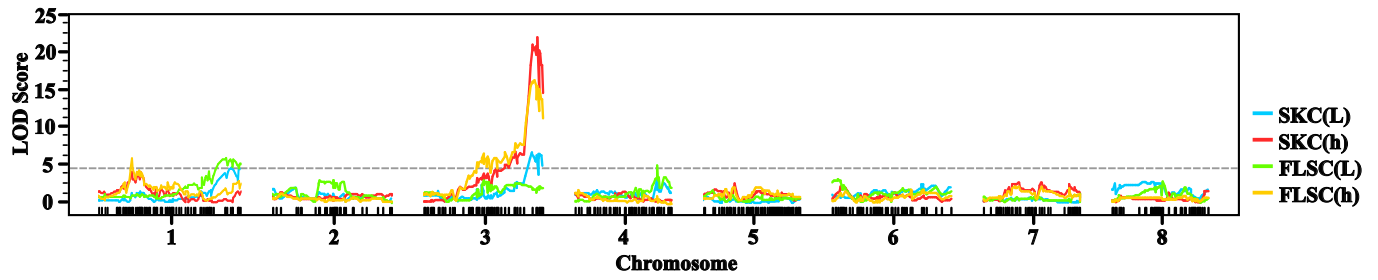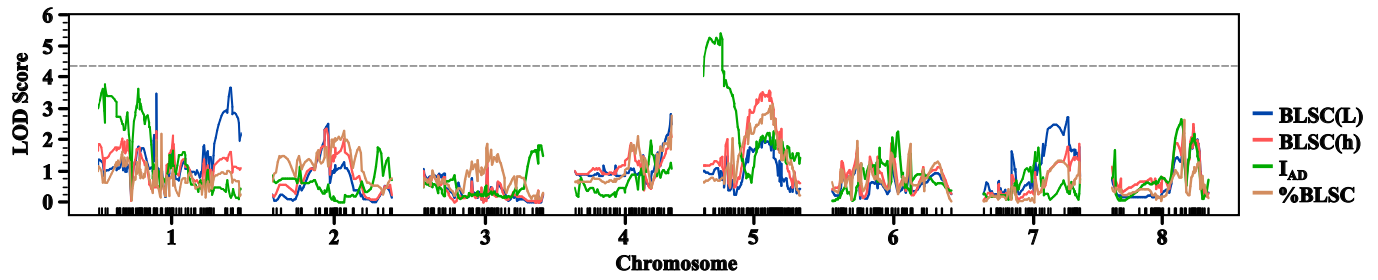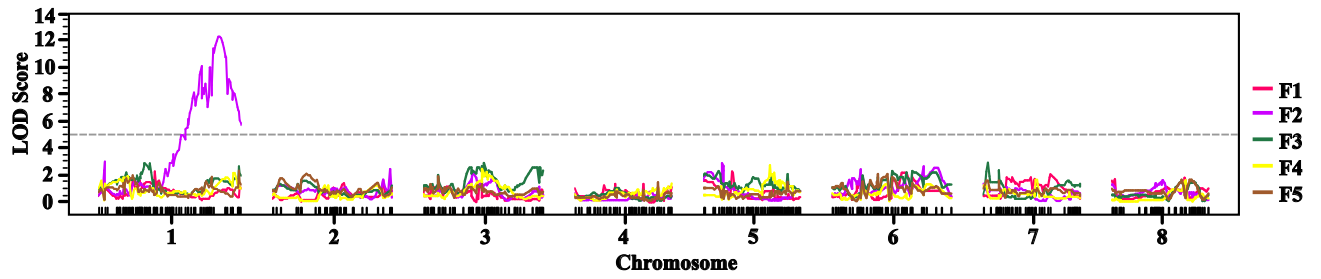

Supplement: Supplementary file 8 — Supplementary Material 8: Figure S3. QTL analysis for the ‘Goldrich’ parent in ‘G × C’ across the entire genetic map for color and flavonoid traits. [file 12864_2026_12989_MOESM8_ESM.pdf]

# QTL ANALYSIS ('CURROT' OF 'GxC')

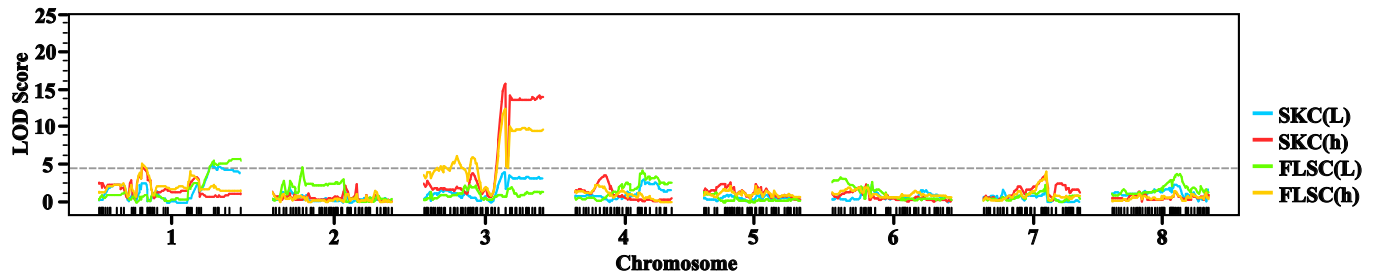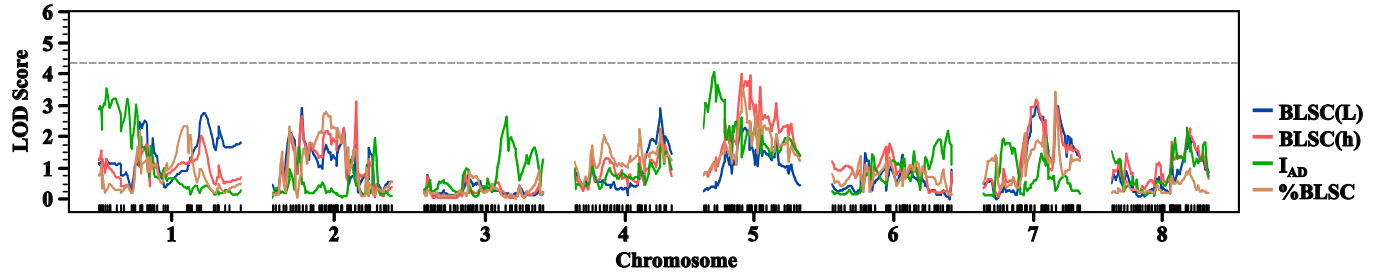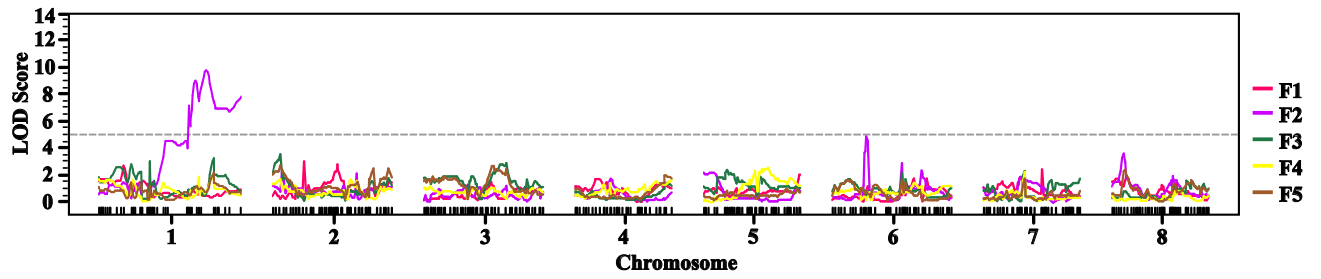

Supplement: Supplementary file 9 — Supplementary Material 9: Figure S4. QTL analysis for the ‘Currot’ parent in ‘B × C’ across the entire genetic map for color and flavonoid traits. [file 12864_2026_12989_MOESM9_ESM.pdf]

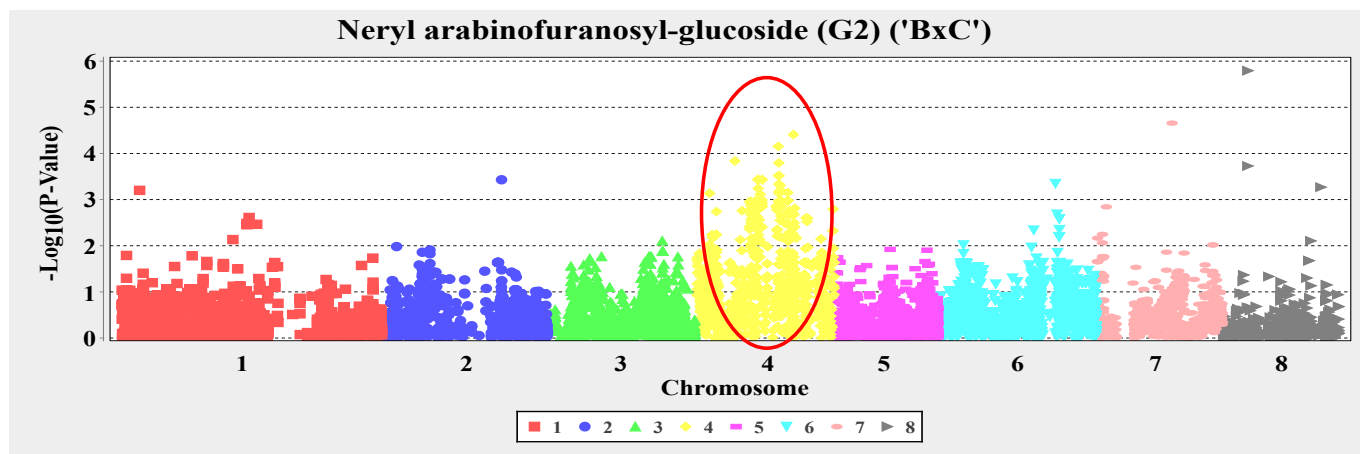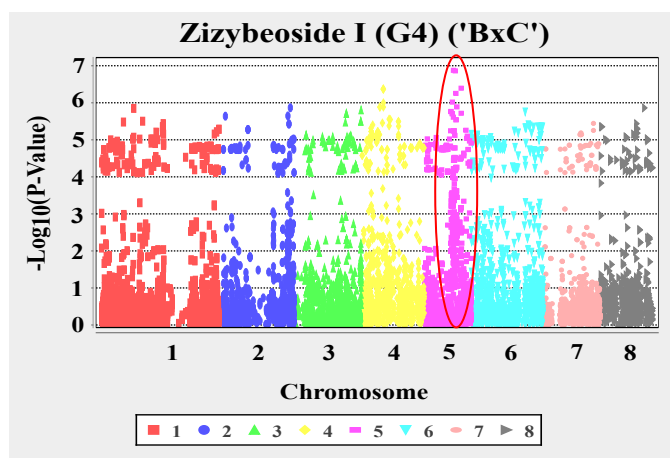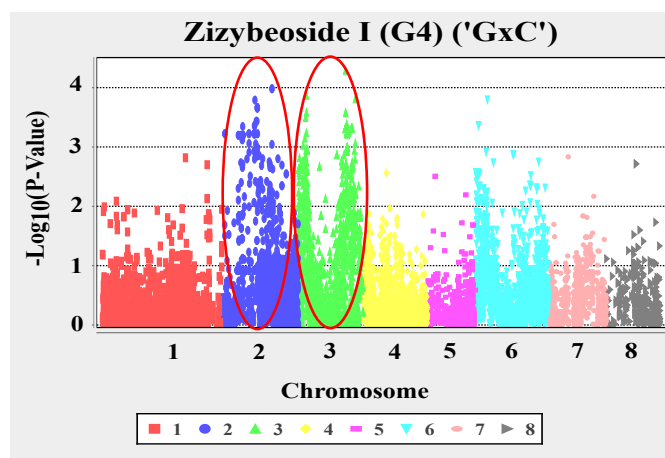

Supplement: Supplementary file 10 — Supplementary Material 10: Figure S5. Above the figure is the Manhattan plot for neryl arabinofuranosyl-glucoside (G2) in the ‘B × C’ population. Below are the Manhattan plots for zizybeoside I (G4) in both the ‘B × C’ and ‘G × C’ populations. The red ellipses highlight the most significant chromosomes in each of the Manhattan plots. [file 12864_2026_12989_MOESM10_ESM.pdf]

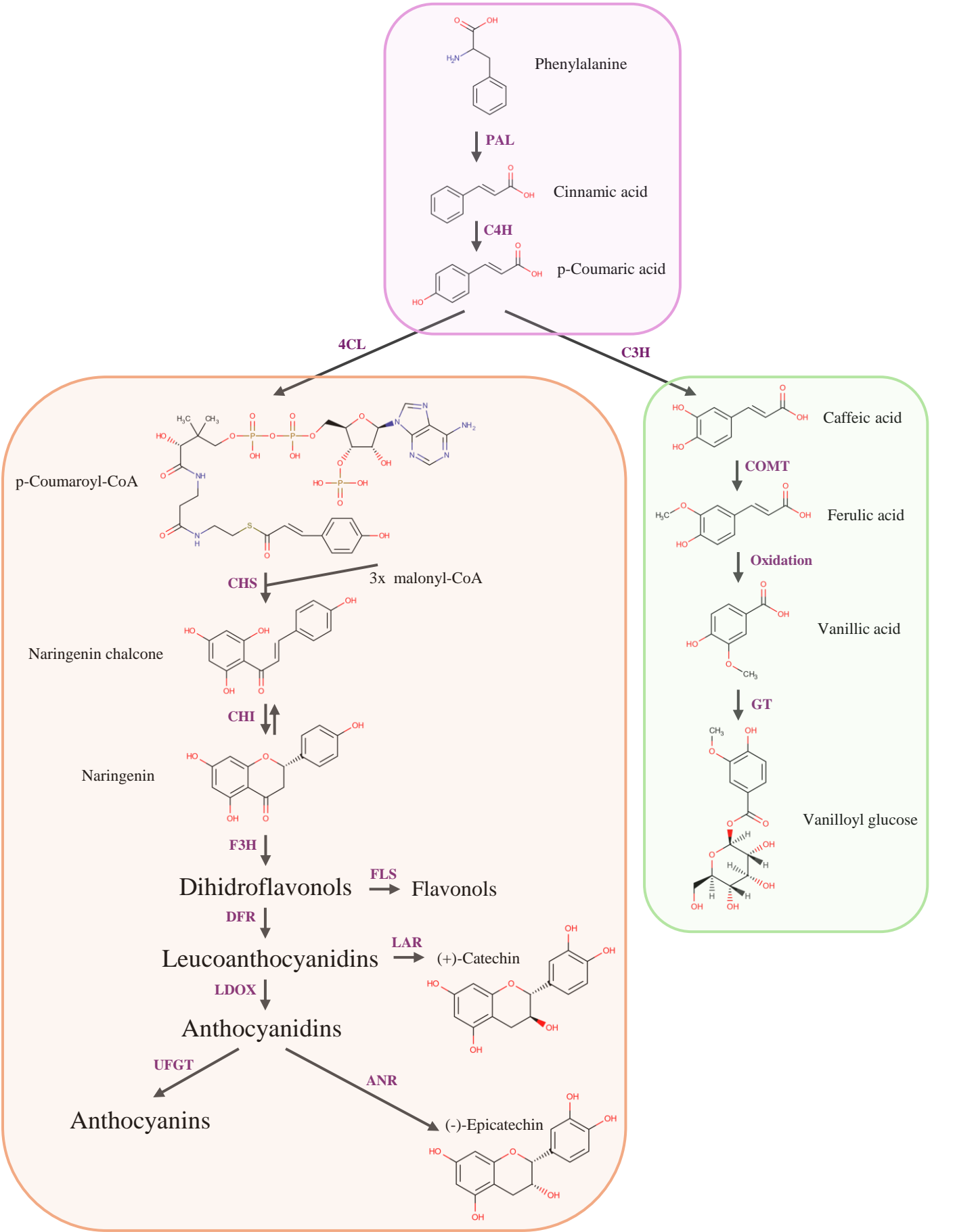

Supplement: Supplementary file 12 — Supplementary Material 12: Figure S7. Biosynthetic pathway of vanilloyl glucose and its interconnection with the phenylpropanoid and anthocyanin pathways. Enzymes and intermediates are depicted to highlight the metabolic flow and regulatory nodes within these interconnected pathways. [file 12864_2026_12989_MOESM12_ESM.pdf]
