# Supplementary material for: Identification of QTLs linked to bioactive flavonoids and glycosides in the apricot fruit (Prunus armeniaca L.)
Source: BMC Genomics. 2026 May 30;27:626. doi: 10.1186/s12864-026-12989-0 (PMC13386964; doi:10.1186/s12864-026-12989-0)
Supplement: Supplementary file 11 — Supplementary Material 11: Figure S6. QTL analysis for each parent in ‘Bergeron’ × ‘Currot’ (‘B × C’) and ‘Goldrich’ × ‘Currot’ (‘G × C’) populations across the entire genetic map for tentatively identified phenolic acids and glycosides. [file 12864_2026_12989_MOESM11_ESM.pdf]

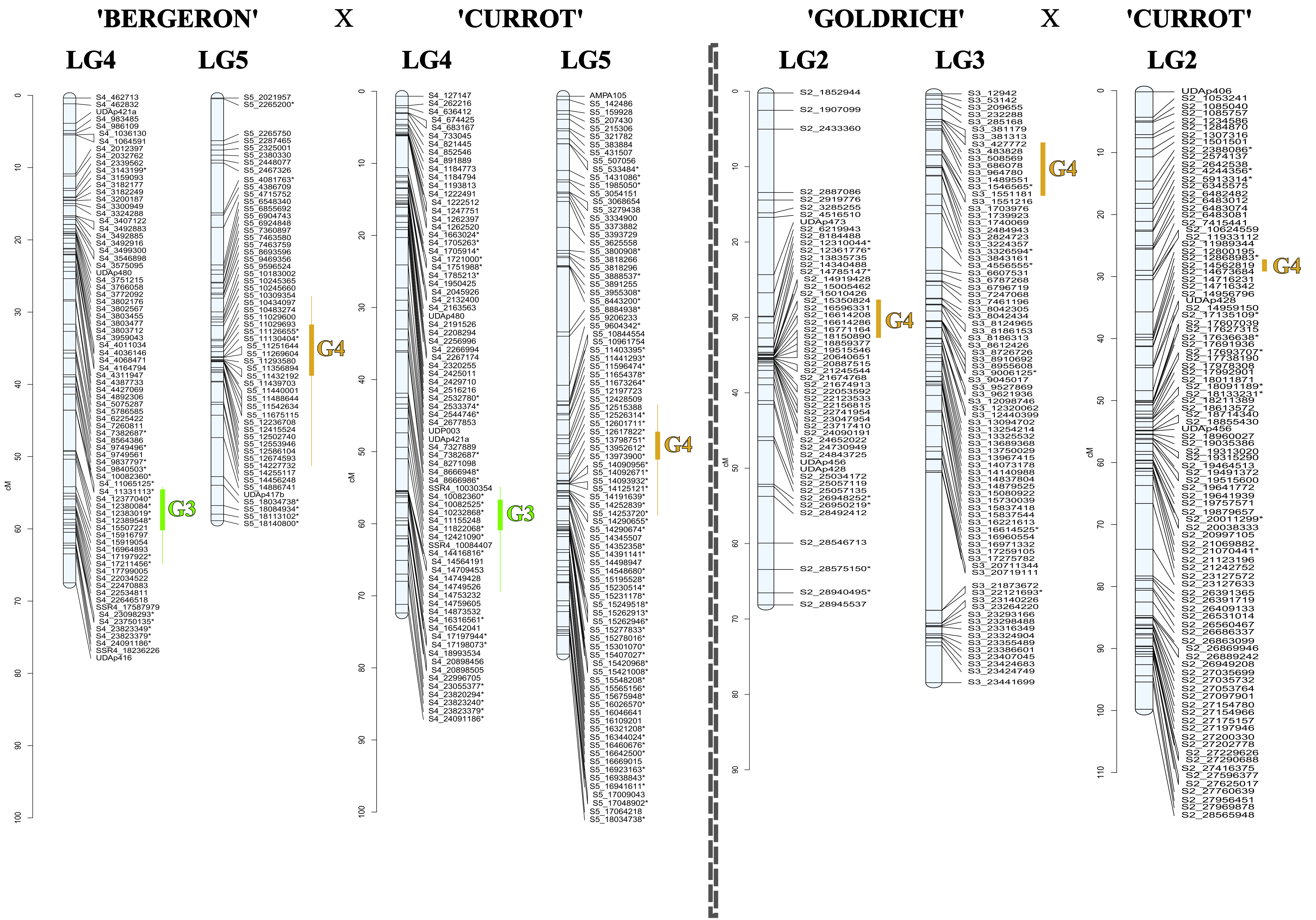
'BERGERON' X 'CURROT'
LG4
LG5
LG4
LG5
'GOLDRICH' X 'CURROT'
LG2
LG3
LG2
cM
G3
G4
G3
G4
G4
G4
G4
